# Impedance-Based Phenotypic Readout of Transporter Function: A Case for Glutamate Transporters

**DOI:** 10.3389/fphar.2022.872335

**Published:** 2022-05-23

**Authors:** Hubert J. Sijben, Laura Dall’ Acqua, Rongfang Liu, Abigail Jarret, Eirini Christodoulaki, Svenja Onstein, Gernot Wolf, Simone J. Verburgt, Sylvia E. Le Dévédec, Tabea Wiedmer, Giulio Superti-Furga, Adriaan P. IJzerman, Laura H. Heitman

**Affiliations:** ^1^ Division of Drug Discovery and Safety, Leiden Academic Centre for Drug Research, Leiden University, Leiden, Netherlands; ^2^ CeMM Research Center for Molecular Medicine of the Austrian Academy of Sciences, Medical University of Vienna, Vienna, Austria; ^3^ Oncode Institute, Leiden, Netherlands

**Keywords:** EAAT, glutamate transporter, solute carrier, label-free, impedance, cell swelling, phenotypic assay

## Abstract

Excitatory amino acid transporters (EAAT/SLC1) mediate Na^+^-dependent uptake of extracellular glutamate and are potential drug targets for neurological disorders. Conventional methods to assess glutamate transport *in vitro* are based on radiolabels, fluorescent dyes or electrophysiology, which potentially compromise the cell’s physiology and are generally less suited for primary drug screens. Here, we describe a novel label-free method to assess human EAAT function in living cells, i.e., without the use of chemical modifications to the substrate or cellular environment. In adherent HEK293 cells overexpressing EAAT1, stimulation with glutamate or aspartate induced cell spreading, which was detected in real-time using an impedance-based biosensor. This change in cell morphology was prevented in the presence of the Na^+^/K^+^-ATPase inhibitor ouabain and EAAT inhibitors, which suggests the substrate-induced response was ion-dependent and transporter-specific. A mechanistic explanation for the phenotypic response was substantiated by actin cytoskeleton remodeling and changes in the intracellular levels of the osmolyte taurine, which suggests that the response involves cell swelling. In addition, substrate-induced cellular responses were observed for cells expressing other EAAT subtypes, as well as in a breast cancer cell line (MDA-MB-468) with endogenous EAAT1 expression. These findings allowed the development of a label-free high-throughput screening assay, which could be beneficial in early drug discovery for EAATs and holds potential for the study of other transport proteins that modulate cell shape.

## 1 Introduction

Glutamate is the main excitatory amino acid in the human central nervous system. Its release from neurons is essential for the activation of ionotropic and metabotropic (mGluR) glutamate receptors in the close vicinity of the release site (Danbolt, 2001). Extracellular concentrations of glutamate are tightly regulated by vesicular release and dedicated solute carrier (SLC) transport proteins that are found on neurons and neighboring glia (Zhou and Danbolt, 2013). The excitatory amino acid transporters (EAAT/SLC1) are the main facilitators of Na^+^-dependent glutamate uptake, with EAAT1 and EAAT2 accounting for roughly 90% of all glutamate uptake in the human central nervous system (Martínez-Lozada and Ortega, 2015). EAAT1 and EAAT2 are mainly expressed on astroglia (Arriza et al., 1994), whereas neuronal cells express EAAT3 (Arriza et al., 1994), EAAT4 (cerebellar Purkinje cells) (Fairman et al., 1995), and EAAT5 (retina) (Arriza et al., 1997). Aberrant function or expression of glutamate transporters has been linked to an extensive list of neurological and psychological disorders, including Alzheimer’s disease (Andersen et al., 2021), Parkinson’s disease (Li et al., 2021), epilepsy (Peterson and Binder, 2020), schizophrenia (Parkin et al., 2018), and depression (Lang and Borgwardt, 2013). Moreover, excessive extracellular concentrations of glutamate are generally linked to excitotoxicity caused by overactivation of glutamate receptors (Danbolt, 2001).

Under most conditions, pharmacological EAAT inhibition leads to highly elevated and detrimental glutamate levels, which refrains this class of modulators from widespread therapeutic applications. As such, most EAAT inhibitors have been mainly developed as probes for mechanistic studies and protein structure elucidations (Shimamoto et al., 2004; Dunlop et al., 2005; Jensen et al., 2009). However, in instances of severe ischemic stroke glutamate transport can be reversed as a result of a disrupted Na^+^/K^+^ balance, effectively increasing the extracellular glutamate levels in which cases EAAT inhibition could be a viable therapeutic strategy (Rossi et al., 2000; Mahmoud et al., 2019). Furthermore, a missense mutation of EAAT1 (P290R) that was identified in a patient with episodic ataxia type 6 shows a gain-of-function of the anion conductivity of EAAT, which contributes to severe ataxia symptoms and cerebellar degeneration (Winter et al., 2012; Kovermann et al., 2020). Another EAAT1 variant (E219D) associated with Tourette’s syndrome has increased plasma membrane insertion probability and elevated glutamate uptake rates (Adamczyk et al., 2011). Thus, EAAT1 inhibition in patients with gain-of-function mutations could be an attractive approach to treat disease. Although so far no EAAT inhibitors have made it into clinical trials, the search for subtype selective modulators that alter the function or expression levels of EAATs is ongoing (Fontana, 2015). Indeed, allosteric enhancers of EAAT2, which increase glutamate uptake, were recently discovered using a hybrid structure-based approach, and could be a potential treatment for excitotoxicity-related diseases (Kortagere et al., 2018). Moreover, the first truly selective EAAT3 inhibitors were described only recently (Wu et al., 2019), indicating that the development of small molecule tools for EAATs is still ongoing and considered an important endeavor.

Several *in vitro* methods are available to screen for EAAT active molecules in endogenous cell lines or cells with heterologous EAAT expression. Traditional electrophysiology approaches are accurate and present kinetic insight in transporter ion fluxes, but are tedious and labor-intensive, which makes them unsuitable for large compound screens (Dvorak et al., 2021). Automated electrophysiology-based methods on solid-supported membranes vastly increase throughput and have been used to study EAAT3 function (Krause et al., 2009; Qiu et al., 2021). Another direct method, uptake of radiolabeled substrate by EAAT-expressing cells provides a rapid and sensitive readout of transporter function and inhibition (Fontana, 2018), although the use and handling of radioactivity may be a drawback to use this method. Alternatively, indirect assays based on fluorescent probes and reporters such as membrane potential dyes (Jensen and Bräuner-Osborne, 2004), glutamate sensors (Armbruster et al., 2020), and intracellular anion sensors (Zielewicz and Grewer, 2019) have proven successful to infer glutamate transport activity, although they require the introduction of non-physiological chemical labels. Recently, we reported on a label-free impedance-based method to assess activity and inhibition of nucleoside (Vlachodimou et al., 2019), dopamine (Sijben et al., 2021a), and norepinephrine transporters (Sijben et al., 2021b) *via* activation of congruent G protein-coupled receptors (GPCRs) by their endogenous substrate in live cells, termed the TRACT assay. Importantly, impedance-based biosensors have the advantage to record any changes in cellular morphology upon cell perturbation, such as receptor activation and acute cytotoxicity (Lundstrom, 2017), which opens a broader application of label-free assays to study transport proteins.

Here, we used an impedance biosensor system, xCELLigence, to study the function of EAATs in a HEK293 cell line with inducible heterologous expression of either one of the five human EAAT subtypes. An in-depth analysis of EAAT1-expressing cells revealed two distinct effects: 1) in cells transfected with the metabotropic glutamate receptor type 2 (mGluR_2_), EAAT1 reduced the apparent potency of glutamate on mGluR_2_, whereas 2) in cells lacking mGluR_2_ glutamate induced EAAT1-mediated, receptor-independent cellular responses. Live-cell imaging revealed that the cells spread upon substrate stimulation, most likely initiated by EAAT1-mediated cell swelling. Substantial transporter-mediated responses were also observed for EAAT2 and EAAT3, but not for EAAT4 and EAAT5, likely due to poor expression of the latter two proteins. In addition, substrate-induced responses could be measured in a cell line with endogenous EAAT1 expression, which together indicate that this phenotypic assay is highly sensitive and applicable to multiple EAAT subtypes. With this method we demonstrate a completely novel approach to study glutamate transporters, effectively expanding the toolbox for mechanistic and drug discovery purposes.

## 2 Materials and Methods

### 2.1 Chemicals and Reagents

Jump In T-REx HEK 293 (JumpIn) overexpressing human EAAT1/2/3/4/5 (see Jump In T-REx HEK 293-EAAT Cell Line Generation section), MDA-MB-468, and 1321N1 cells were kindly provided by the RESOLUTE consortium (Research Center for Molecular Medicine, Medical University of Vienna, Austria). L-glutamic acid monosodium salt monohydrate (L-glu), L-aspartic acid monosodium salt monohydrate (L-asp), D-glutamic acid (D-glu), D-aspartic acid (D-asp), doxycycline hyclate and ouabain octahydrate were purchased from Sigma Aldrich (St. Louis, MO, United States). 2-amino-4-(4-methoxyphenyl)-7-(naphthalen-1-yl)-5-oxo-5,6,7,8-tetrahydro-4H-chromene-3-carbonitrile (UCPH-101) was purchased from Santa Cruz Biotechnology (Dallas, TX, United States). (2S, 3S)-3-[3-[4-(trifluoromethyl)benzoylamino]benzyloxy] aspartate (TFB-TBOA) was purchased from Axon Medchem (Groningen, Netherlands). (2S)-2-amino-2-[(1S, 2S)-2-carboxycycloprop-1-yl]-3-(xanth-9-yl) propanoic acid (LY341495) was purchased from Cayman Chemical (Ann Arbor, MI, United States). xCELLigence PET E-plates 96 (ACEA Biosciences, San Diego, CA, United States) were purchased from Bioké (Leiden, Netherlands). cDNA encoding the human metabotropic glutamate receptor 2 (*GRM2*, ORF: NM_000839) containing an N-terminal FLAG-tag cloned in a pcDNA3.1 (+) plasmid (mGluR_2_ cDNA), as well as empty pcDNA3.1 (+) plasmid (mock cDNA) were purchased from GenScript (Piscataway, NJ, United States). The lentiviral GFP-LifeAct cDNA expression vector was provided by Dr. Olivier Pertz (University of Basel, Basel, Switzerland). Puromycin was purchased from Acros Organics/Fisher Scientific (Landsmeer, Netherlands). PNGaseF was purchased from New England Biolabs (Ipswich, MA, United States). Pierce ECL Western blotting substrate was purchased from Thermo Fisher Scientific (Waltham, MA, United States). All other chemicals were of analytical grade and obtained from standard commercial sources.

### 2.2 JumpIn-EAAT Cell Line Generation

JumpIn cells with doxycycline (dox)-inducible transgene overexpression were generated for either one of the five human EAAT subtypes by the RESOLUTE consortium. JumpIn cells were cultured and transfected as described previously (Sijben et al., 2021a; 2021b). In short, cells were cultured in culture medium supplemented with 200 μg/ml hygromycin B and 5 μg/ml blasticidin. For transfection, a codon-optimized ORF for either human EAAT1 (*SLC1A3*, Addgene #131889), EAAT2 (*SLC1A2*, Addgene #131901), EAAT3 (*SLC1A1*, Addgene #131878), EAAT4 (*SLC1A6*, Addgene #131986) or EAAT5 (*SLC1A7*, Addgene #131998) was cloned into a Gateway-compatible pJTI R4 DEST CMV TO pA expression vector with a C-terminal Twin-Strep-tag followed by an HA-tag. Successfully transfected cells were selected in culture medium supplemented with 2 mg/ml geneticin (G418) and 5 μg/ml blasticidin and were subsequently pooled for use in further experiments. Expression of the transgene was induced by incubating the cells for 24 h in presence of 1 μg/ml dox.

### 2.3 Cell Culture

JumpIn-EAAT cells were cultured as adherent cells in high glucose Dulbecco’s Modified Eagle’s Medium (DMEM) containing 10% (v/v) fetal calf serum (FCS), 2 mM Glutamax, 100 IU/ml penicillin and 100 μg/ml streptomycin (culture medium) at 37°C and 5% CO_2_. Cells were thawed and split for 1–2 passages in culture medium before switching to selection medium (culture medium supplemented with 2 mg/ml G418 and 5 μg/ml blasticidin) for up to 1 week. Subsequently, the cells were maintained in culture medium at least 24 h prior to performing an experiment. Cells were split twice weekly at 1:8–1:16 ratios in 10 cm culture dishes.

MDA-MB-468 cells were cultured as adherent cells in DMEM containing 10% (v/v) FCS, 2 mM Glutamax, 100 IU/ml penicillin and 100 μg/ml streptomycin (culture medium) at 37°C and 5% CO_2_. Cells were split twice weekly at 1:3–1:4 ratios in 10 cm culture dishes.

### 2.4 Transient Transfection of JumpIn-EAAT1 Cells

JumpIn-EAAT1 cells were transiently transfected using 25 kDa linear polyethyleneimine (PEI) as a transfection reagent (Boussif et al., 1995). Cells were split in 10 cm dishes to achieve ∼60% confluence on the day of transfection. A mix of 7.5 μg/ml PEI and 2.5 µg total cDNA (mock or mGluR_2_) in 1 ml Opti-MEM was incubated at room temperature for 30 min. Subsequently, cells were refreshed in penicillin/streptomycin-free culture medium before adding the PEI:DNA mix to each dish. Cells were incubated for 24 h at 37°C and 5% CO_2_ prior to use in the TRACT assay.

### 2.5 JumpIn-EAAT1-LifeAct-GFP Cell Line Generation

For visualization of the actin cytoskeleton, JumpIn-EAAT1 cells were transduced with a lentiviral GFP-LifeAct cDNA expression vector and were maintained in culture medium containing 2 μg/ml puromycin to select for successfully transduced cells.

### 2.6 xCELLigence Assays

#### 2.6.1 General

Label-free whole-cell assays, based on cell-induced changes in impedance, were performed using the xCELLigence real-time cell analyzer (RTCA) system (ACEA Biosciences, San Diego, CA, United States) as described previously (Vlachodimou et al., 2019; Sijben et al., 2021a, 2021b). In short, cells are cultured in medium on gold-plated electrodes in microwell E-plates. Per well impedance is continuously measured at 10 kHz and is converted to the unitless parameter Cell Index (CI) by the following formula:

$$CI=\frac{\left(Z_{i}-Z_{0}\right)\text{Ω}}{15\text{Ω}}$$

where Z_i_ is the impedance at any given time point and Z_0_ is the baseline impedance measured at the start of each experiment (Kho et al., 2015).

Assays were performed at 37°C and 5% CO_2_ in 96-well PET E-plates in a total volume of 100 µl per well. Prior to cell seeding, the baseline impedance (Z_0_) was measured in the recording station in 40 µl (two compound additions) or 45 µl (one compound addition) medium in the presence (+) or absence (−) of 1 μg/ml dox. All compounds were diluted in phosphate-buffered saline (PBS) and added in 5 µl per addition using a VIAFLO 96 handheld electronic 96 channel pipette (INTEGRA Biosciences, Tokyo, Japan). When DMSO was used as a solvent for a compound, the final amount of DMSO was kept at 0.1% per well and was included in the vehicle (PBS/DMSO). All conditions were tested at two technical replicates per plate.

#### 2.6.2 Assay Procedures

On the day of the experiment, JumpIn-EAAT cells grown to 70–80% confluence were trypsinized (0.25% trypsin in PBS/EDTA), counted and seeded in the E-plate in a volume of 50 μl at 60,000 cells/well. Transiently transfected JumpIn-EAAT1 cells were detached using only PBS/EDTA. The E-plate was left to rest at room temperature for 30 min prior to replacement in the recording station at 37°C and 5% CO_2_. Cell growth was recorded overnight for 22 h. If a pretreatment was required for the experiment, the cells were pretreated after 22 h with either a concentration of inhibitor or vehicle (PBS/DMSO) and cells were monitored for 60 min. For GPCR antagonist experiments, a maximum concentration of LY341495 (1 µM) was used. For EAAT inhibition experiments increasing concentrations (1 nM–10 µM) of UCPH-101 and TFB-TBOA were used. After the pretreatment, cells were stimulated with substrate or vehicle (PBS). For GPCR antagonist and EAAT inhibition experiments, cells were stimulated with a submaximal concentration of L-glu, i.e., 100 μM L-glu with mGluR_2_ antagonist, 1 mM L-glu for EAAT1 inhibition and 316 μM L-glu for EAAT2 and EAAT3 inhibition. After stimulation the impedance was measured for at least 2 h. Following each experiment, the cells were washed from the E-plates using sterile H_2_O and 70% ethanol and the plates were sterilized for 1 h under UV light (Stefanowicz-Hajduk et al., 2016; Xu et al., 2017). Properly washed plates were re-used up to two times.

#### 2.6.3 High-Throughput Screening Validation

The impedance-based phenotypic assay for JumpIn-EAAT1 cells was assessed for reproducibility and robustness in a semi-manual high-throughput screening (HTS) validation as described previously (Sijben et al., 2021b). Briefly, three 96-well E-plates were run consecutively on 1 day for 3 days in a row. Each plate consisted of alternating columns of wells producing a high, mid or low signal, which were JumpIn-EAAT1 cells pretreated with either vehicle (PBS/DMSO), 0.2 µM or 10 µM TFB-TBOA, respectively. After 1 h pretreatment, cells were stimulated with 1 mM L-glutamate. The assay procedure was the same as for the regular xCELLigence assays.

### 2.7 Automated Microscopy

JumpIn-EAAT1-LifeAct-GFP cells were seeded at 20,000 cells/well on a SCREENSTAR black-walled clear-bottom 96-well culture plate in 90 µl culture medium and grown for 24 h at 37°C and 5% CO_2_. Cells were pretreated for 1 h with PBS/DMSO and subsequently stimulated with vehicle (PBS) or 1 mM L-glutamate upon which cells were immediately imaged using automated microscopy. Microscopy was performed on a Nikon Eclipse Ti2 C2+ confocal microscope (Nikon, Amsterdam, Netherlands). This system was equipped with a 37°C incubation chamber, an automated xy-stage, an integrated Perfect Focus System (PFS) and 408, 488, and 561 nm lasers. The system was controlled by Nikon’s NIS software. All images were acquired using a Plan-Apochromat ×20 objective with 0.75 NA, at a resolution of 512 × 512 pixels.

### 2.8 Targeted Metabolomics

#### 2.8.1 Metabolite Extraction

JumpIn-EAAT1 cells were plated at 150,000 cells/well in 24 well poly-L-lysine coated plates in culture medium in the absence (−dox) or presence (+dox) of 1 μg/ml doxycycline and grown at 37°C and 5% CO_2_. After 24  h, the medium was refreshed and cells were pretreated with vehicle (PBS/DMSO) or 10 µM TFB-TBOA. After 1 h pretreatment, cells were stimulated for 2 h with vehicle (PBS) or 1 mM L-glutamate or L-aspartate. For lysis, cells were first gently washed with room temperature bicarbonate buffer (91 mM NH_4_HCO_3_, pH 7.4). Then, cells were transferred to ice where 300 µl/well of ice-cold 80:20 MeOH:H_2_O containing a mixture of isotopically labeled internal standards [Metabolite Yeast Extract (U-13C, 98%), ISOtopic Solutions, Vienna, Austria] was added to each well. The cells were then scraped and transferred to a pre-cooled Eppendorf tube and immediately snap frozen in liquid nitrogen. Samples were thawed on ice before being centrifuged at 16,000 × g for 10 min at 4°C. The clarified metabolite-containing supernatants were moved into a high-performance liquid chromatography vial and stored at −80°C until LC-MS/MS analysis.

#### 2.8.2 LC-MS/MS Analysis

Cell extracts were dried using a nitrogen evaporator. The dry residue was reconstituted in 16 µl H_2_O and 4 µl of sample extract was used for LC-MS/MS analysis. A 1290 Infinity II UHPLC system (Agilent Technologies) coupled to a 6470 triple quadrupole mass spectrometer (Agilent Technologies) was used for the LC-MS/MS analysis. The chromatographic separation for samples was carried out on a ZORBAX RRHD Extend-C18, 2.1 × 150 mm, 1.8 µm analytical column (Agilent Technologies). The column was maintained at a temperature of 40°C and 4 µl of sample was injected per run. The mobile phase A was 3% MeOH (v/v), 10 mM tributylamine, 15 mM acetic acid in H_2_O, and mobile phase B was 10 mM tributylamine, 15 mM acetic acid in MeOH. The gradient elution with a flow rate 0.25 mL/min was performed for a total time of 24 min. Afterward, a back flushing of the column using a 6 port 2-position divert valve was carried out for 8 min using acetonitrile, followed by 8 min of column equilibration with 100% mobile phase A. The triple quadrupole mass spectrometer was operated in an electrospray ionization negative mode, spray voltage 2 kV, gas temperature 150°C, gas flow 1.3 L/min, nebulizer 45  psi, sheath gas temperature 325°C, and sheath gas flow 12 L/min. The metabolites of interest were detected using a dynamic Multiple Reaction Monitoring (MRM) mode. The MassHunter 10.0 software (Agilent Technologies) was used for the data processing. Ten-point linear calibration curves with internal standardization were constructed for the quantification of metabolites.

### 2.9 Western Blot Analysis

JumpIn-EAAT cells were grown at 1 × 10^6^ cells/well on a 6-well culture plate for 24 h in the presence or absence of 1 μg/ml doxycycline at 37°C and 5% CO_2_. Cells were lysed in lysis buffer [50 mM HEPES (pH 7.4), 250 mM NaCl, 5 mM EDTA, 1% NP-40, 1 tablet complete EDTA-free protease inhibitor cocktail (Roche) per 50 ml] for 30 min on ice and centrifuged at 20,800 × g, 4°C for 15 min. Protein concentration was measured using the bicinchoninic acid (BCA) protein assay (Smith et al., 1985). Lysates were either treated or not treated with PNGaseF overnight at 37°C and samples were subsequently denatured and separated by SDS-PAGE, then transferred to nitrocellulose membranes. The membranes were blocked in 5% milk + TBS-T for 1 h and cut at 25 kDa. The top membranes (>25 kDa) were incubated with horse radish peroxidase (HRP)-conjugated anti-HA-7 (Sigma, H6533, 1:7000) and bottom membranes (<25 kDa) with cyclophilin B antibody (Abcam, ab178397, 1:5000) in 5% milk + TBS-T overnight at 4°C. The bottom membrane was subsequently incubated with HRP-conjugated anti-rabbit IgG (Jackson ImmunoResearch, 111-035-003, 1:5000) in 5% milk + TBS-T for 1 h at 4°C. Both membranes were visualized using Pierce ECL substrate.

### 2.10 Data Analysis

#### 2.10.1 xCELLigence

Experimental data was recorded using RTCA Software v2.0 or v2.1.1 (ACEA Biosciences). In the RTCA Software CI values were normalized to the time point prior to substrate/agonist stimulation resulting in normalized Cell Index (nCI) values. The nCI data were exported and subsequent analyses and data visualizations were done using GraphPad Prism v9 (GraphPad Software, San Diego, CA, United States). In all experiments, nCI values of vehicle-only conditions were subtracted from all other conditions to correct for vehicle-induced, substrate-independent effects. The resulting nCI responses were quantified by taking the net area under the curve (AUC) of the first 120 min after substrate/agonist stimulation, unless stated otherwise. The potency values of substrates and inhibitory potencies of EAAT inhibitors were determined by fitting the net AUC data to a sigmoidal concentration-effect curve with a variable slope.

#### 2.10.2 High-Throughput Screening Validation

For intra-plate variability tests, the net AUC of non-corrected nCI values were used to determine the signal window (SW, indicating dynamic range of the signal) using the following formula (Iversen et al., 2004):

$$SW=\frac{\left(AVG_{high}-\frac{3SD_{high}}{\sqrt{n}}\right)-\left(AVG_{low}+\frac{3SD_{low}}{\sqrt{n}}\right)}{\frac{SD_{high}}{\sqrt{n}}}$$

where *n* is the number of technical replicates per compound in the intended screening assay (e.g., for duplicate measurements *n* = 2), AVG is the average and SD is the standard deviation of the AUC of the high or low signal. Similarly, the Z′ factor (Z′, indicating separation of the high and low signals) is calculated using the following formula (Zhang et al., 1999; Iversen et al., 2004):

$$Z\prime =\frac{\left(AVG_{high}-\frac{3SD_{high}}{\sqrt{n}}\right)-\left(AVG_{low}+\frac{3SD_{low}}{\sqrt{n}}\right)}{\left(AVG_{high}-AVG_{min}\right)}$$

The reported SW and Z′ are the mean ± SEM of all nine E-plates. According to Iversen et al.(2004), the recommended acceptance criterion for an HTS amenable assay is a SW ≥ 2 or Z′ ≥ 0.4.

#### 2.10.3 Targeted Metabolomics

Volcano plots were generated to visualize the significant up- or downregulation of intracellular metabolite concentrations. The log2 fold change upon substrate addition is plotted against the −log10 adjusted *p*-value. A Benjamini-Hochberg correction (Benjamini and Hochberg, 1995) was used to reduce the false discovery rate to obtain the adjusted *p*-value.

#### 2.10.4 Statistics

Data are shown as mean ± standard error of the mean (SEM) of at least three separate experiments each performed in duplicate, unless stated otherwise. Significant difference between two mean potency values was determined by an unpaired two-tailed Student’s t-test. Comparison of multiple mean values to each other or a vehicle control was done using a one-way ANOVA with Tukey’s post-hoc test or Dunnett’s post-hoc test, respectively. Comparison of multiple mean values between two data sets was done using a two-way ANOVA with Šídák’s post-hoc test. Differences were considered statistically significant when *p*-values were below 0.05.

## 3 Results

### 3.1 L-Glutamate Induces Distinct mGluR_2_- and EAAT1-Mediated Responses in a TRACT Assay

**FIGURE 1:**
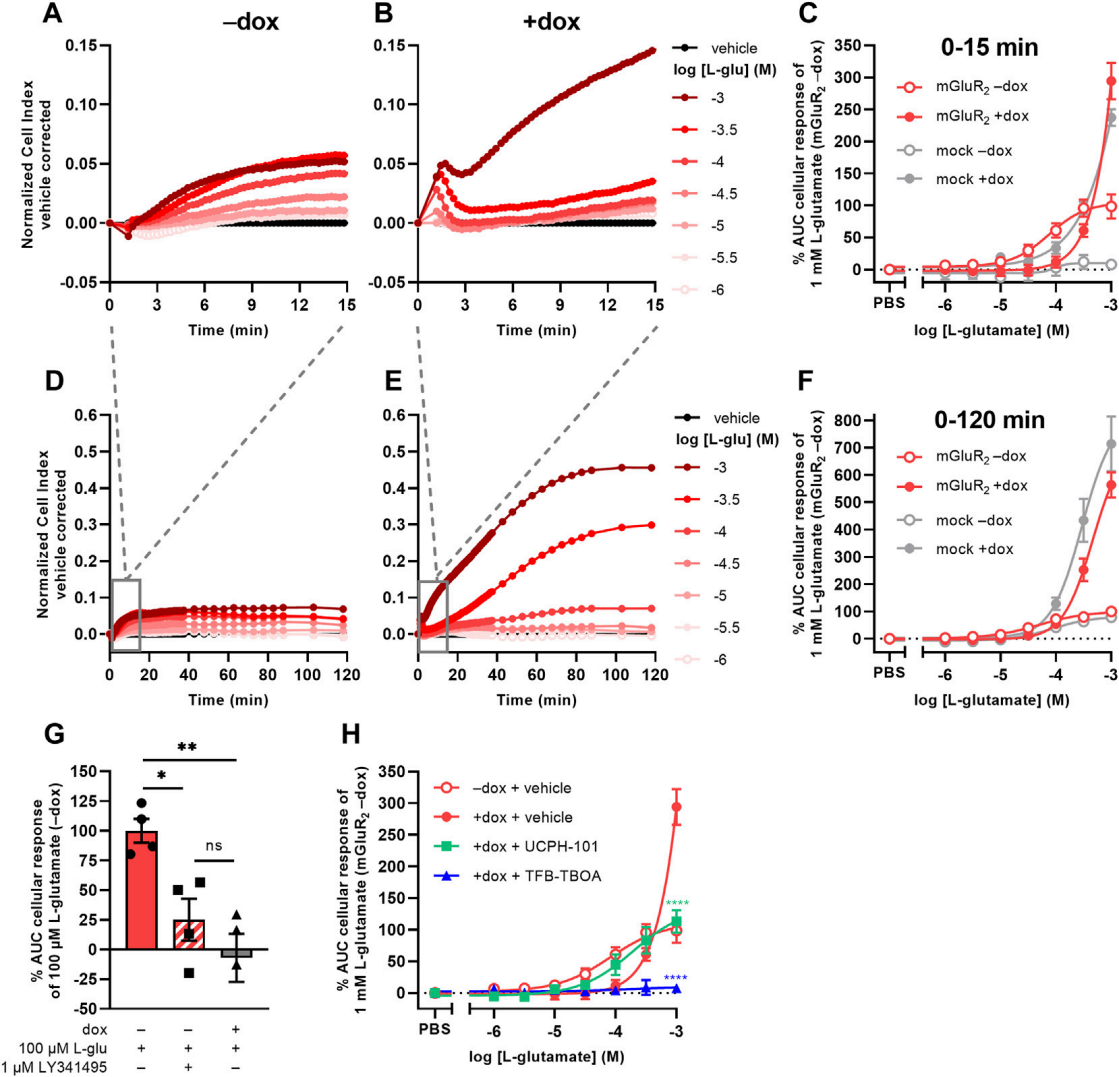
L-glutamate induces distinct mGluR2- and EAAT1-mediated responses in a TRACT assay. **(A,B,D,E)** Vehicle-corrected normalized Cell Index (nCI) traces of the first 15 min **(A,B)** or 120 min **(D,E)** after stimulation of cells with vehicle (PBS) or L-glutamate (L-glu) in the absence (−dox) **(A,D)** or presence (+dox) **(B,E)** of doxycycline, shown as the mean of a representative experiment performed in duplicate. **(C,F)** Combined concentration-response curves of L-glu on JumpIn-EAAT1-mGluR_2_ cells (±dox, red) and mock-transfected JumpIn-EAAT1 cells (mock) (±dox, grey). **(G)** Cellular response of 100 μM L-glu on JumpIn-EAAT1-mGluR_2_ cells (±dox) pretreated for 1 h with vehicle (PBS/DMSO) or 1 µM LY341495. **(H)** Combined concentration-response curves of L-glu on JumpIn-EAAT1-mGluR_2_ cells (±dox) pretreated for 1 h with vehicle (PBS/DMSO) (data derived from Panel 1C), 10 µM UCPH-101 or 10 µM TFB-TBOA. Cellular response is expressed as the net AUC of the first 15 min **(C,G,H)** or 120 min **(F)** after L-glu stimulation. Data are normalized to the response of 1 mM **(C,F,H)** or 100 µM **(G)** L-glu on JumpIn-EAAT1-mGluR_2_ (−dox) cells. Data are shown as the mean ± SEM of three or four individual experiments each performed in duplicate. ns = not significant, *p < 0.05, **p < 0.01, ***p < 0.001, ****p < 0.0001; one-way ANOVA with Tukey’s post-hoc test **(G)** or two-way ANOVA with Šídák’s post-hoc test compared to vehicle-treated +dox cells **(H)**.

Initial attempts to set up a functional method for EAATs were focused on the use of an impedance-based TRACT assay, i.e., using receptor activation as a measure of transporter activity (Sijben et al., 2021a; 2021b). To validate this approach we used a modified HEK293 cell line with doxycycline (dox)-inducible overexpression of EAAT1 (JumpIn-EAAT1) that was transiently transfected with a plasmid encoding metabotropic glutamate receptor type 2 (mGluR_2_) (JumpIn-EAAT1-mGluR_2_). In the TRACT assay, the changes in electrical impedance are reported as the unitless parameter Cell Index (CI), which increases or decreases as the electrode coverage by cells increases or decreases, respectively. In non-induced (−dox) cells L-glutamate (L-glu) induced a concentration-dependent increase of the normalized Cell Index (nCI) within 15 min after stimulation (pEC_50_ = 4.1 ± 0.1) (Figures 1A,C, Table 1), where a plateau was maintained for at least 120 min (Figure 1D). The early-phase L-glu response was attributed to mGluR_2_ activation, as the response of a submaximal concentration of L-glu could be significantly blocked (*p* = 0.0265) by the mGluR_2_-specific antagonist LY341495 (Figure 1G). No distinct early-phase response was observed in non-induced, mock-transfected cells (Figure 1C).

**TABLE 1:** List of (−log) potency (pEC_50_) and inhibitory potency (pIC_50_) values of EAAT substrates and inhibitors on various cell lines in the TRACT and phenotypic assays. Potencies were determined on non-induced (−) or doxycycline (dox)-induced (+) cells in the absence (−) or presence of an inhibitor by analyzing the net area under the curve (AUC) of the first 120 min after substrate stimulation (unless stated otherwise). pIC_50_ values are written in italic. N.D. = not determined.

Assay	Cell line	Dox	Substrate	Inhibitor	pEC_50_ ± SEM	*n*
					pIC_50_ ± SEM	
TRACT assay	**Substrate pEC_50_ ± SEM (AUC = 0–15 min)**					
	JumpIn-EAAT1-mGluR_2_	−	L-glutamate	−	4.1 ± 0.1	6
		+	L-glutamate	−	<3.0	7
		+	L-glutamate	UCPH-101^a^	3.7 ± 0.2	3
		+	L-glutamate	TFB-TBOA^a^	N.D.	3
		JumpIn-EAAT1-mock	−	L-glutamate	−	N.D.	3
	+		L-glutamate	−	<3.0	3
	**Substrate pEC_50_ ± SEM (AUC = 0–120 min)**					
	JumpIn-EAAT1-mGluR_2_	−	L-glutamate	−	4.3 ± 0.1	6
		+	L-glutamate	−	3.3 ± 0.1	7
			JumpIn-EAAT1-mock	−	L-glutamate	−	4.0 ± 0.1	3
	+			L-glutamate	−	3.5 ± 0.0	3
	Phenotypic assay	**Substrate pEC_50_ ± SEM**					
JumpIn-EAAT1		−	L-glutamate	−	4.2 ± 0.3	8
		+	L-glutamate	−	3.4 ± 0.0	10
		+	D-glutamate	−	<3.0	5
		+	L-aspartate	−	3.4 ± 0.0	6
		+	D-aspartate	−	3.4 ± 0.1	5
		JumpIn-EAAT2	−	L-glutamate	−	3.8 ± 0.1	6
+			L-glutamate	−	3.6 ± 0.0	6
JumpIn-EAAT3			−	L-glutamate	−	N.D.	6
		+	L-glutamate	−	3.9 ± 0.0	6
		MDA-MB-468^b^	−	L-glutamate	−	4.0 ± 0.2^b^	3
**Inhibitor pIC_50_ ± SEM (M)**					
JumpIn-EAAT1		+	L-glutamate	Ouabain	7.2 ± 0.0^c^	3
			+	L-glutamate	UCPH-101	5.2 ± 0.1^c^	7
			+	L-glutamate	TFB-TBOA	6.7 ± 0.1^c^	6
				JumpIn-EAAT2	+	L-glutamate	TFB-TBOA	7.1 ± 0.0^d^	3
		JumpIn-EAAT3			+	L-glutamate	TFB-TBOA	6.1 ± 0.2^d^	3

a 10 µM inhibitor.

b AUC = 0–180 min.

c stimulated with 1 mM L-glutamate.

d stimulated with 316 μM L-glutamate.

In cells with dox-induced EAAT1 expression (+dox) stimulation with L-glu resulted in a sharp nCI increase within 2 min followed by a brief decrease and a subsequent gradual increase in nCI in the first 15 min (pEC_50_ < 3.0) reaching a plateau after approximately 120 min that was 6-fold higher than in non-induced cells (Figures 1B,C,E,F, Table 1). Interestingly, 1 mM L-glu produced a vastly elevated nCI response within the first 15 min in dox-induced cells (Figure 1B), whereas at concentrations between 10 and 316 µM the L-glu response was lower than in non-induced cells (Figure 1C), indicating that EAAT1 lowers the extracellular L-glu concentration leading to reduced mGluR_2_ activation. In dox-induced mock-transfected cells the L-glu response over 120 min was in line with JumpIn-EAAT1-mGluR_2_ (Figures 1C,F), which suggests that the L-glu response was comprised of two distinct phases: an early mGluR_2_-dependent phase (0–15 min) and a late EAAT1-mediated phase (0–120 min).

The role of EAAT1 in the early-phase mGluR_2_ response was confirmed using the non-competitive selective EAAT1 inhibitor UCPH-101 (Jensen et al., 2009) and competitive non-selective EAAT inhibitor TFB-TBOA (Shimamoto et al., 2004) in JumpIn-EAAT1-mGluR_2_ cells (Figure 1H). Of note, addition of TFB-TBOA on its own resulted in a peak nCI after 15 min in both non-induced and dox-induced cells, whereas UCPH-101 induced a response in non-induced cells only (Supplementary Figures S1A–D). In dox-induced cells UCPH-101 substantially enhanced the apparent potency of L-glu (pEC_50_ = 3.7 ± 0.2) compared to vehicle-pretreated cells, indicating that EAAT1 inhibition potentiates mGluR_2_ activation (Figure 1H, Table 1). The maximal response of 1 mM L-glu was significantly (*p* < 0.0001) reduced by 62% in the presence of UCPH-101, confirming that this part of the response is EAAT1-mediated. Strikingly, TFB-TBOA completely blocked 316 µM (*p* = 0.0769) and 1 mM L-glu (*p* < 0.0001) responses. Since differential effects of the two EAAT1 inhibitors on the mGluR_2_ response complicated data interpretation, which is impractical for a screening assay, we did not pursue optimization of the TRACT assay. Rather, we further explored the late-phase EAAT1-mediated L-glu response.

### 3.2 EAAT1-Specific L-Glutamate-Induced Cellular Responses are Blocked by UCPH-101 and TFB-TBOA

**FIGURE 2:**
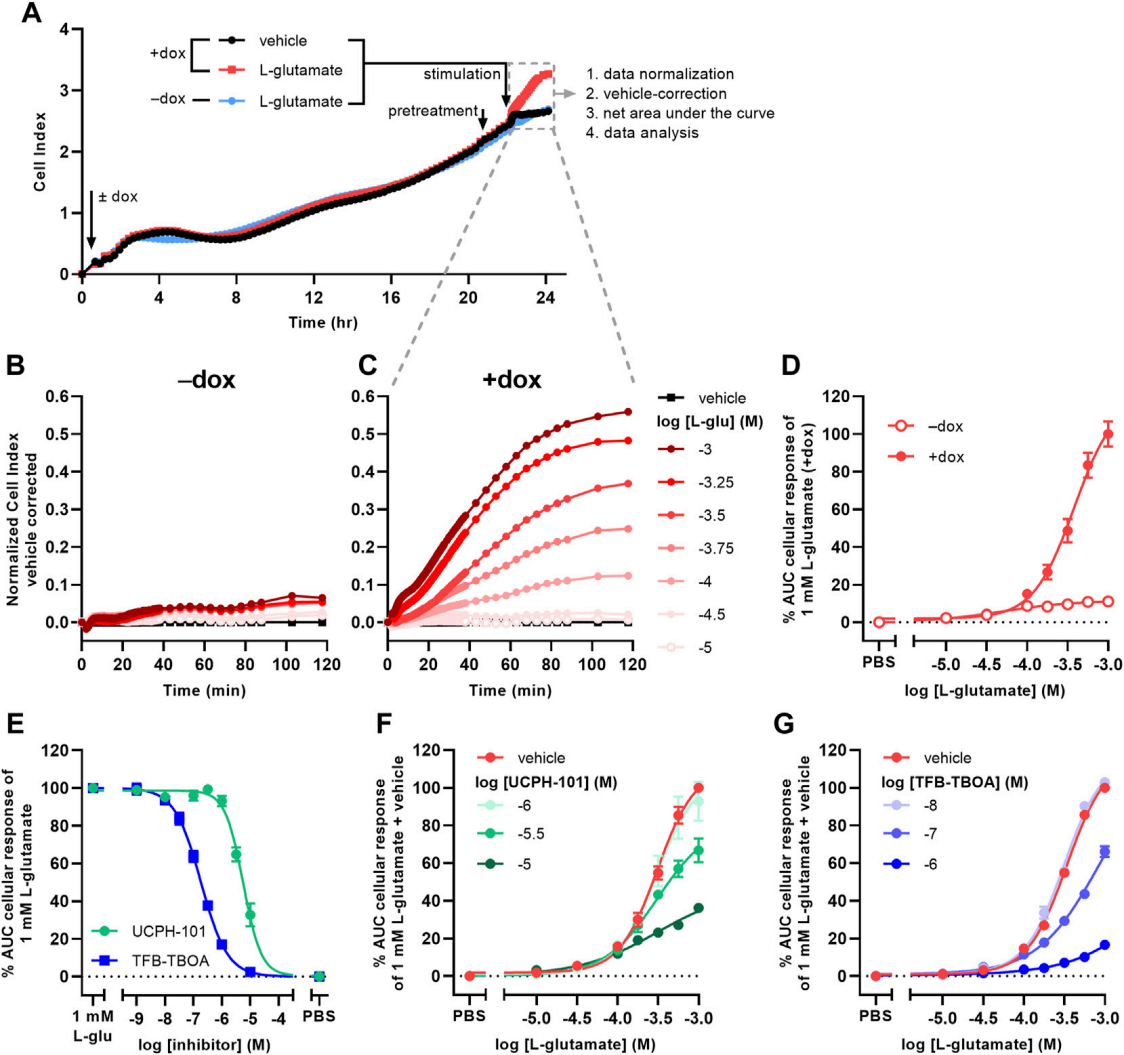
L-glutamate induces EAAT1-specific cellular responses on JumpIn-EAAT1 cells. **(A)** Experimental layout of an xCELLigence growth curve, the subsequent assay course and data analysis. Shown are traces of two separate wells from a representative experiment. **(B,C)** Vehicle-corrected nCI traces of cells in the absence (−dox) **(B)** or presence (+dox) **(C)** of doxycycline stimulated with vehicle (PBS) or L-glutamate (L-glu), shown as the mean of a representative experiment performed in duplicate. **(D)** Combined concentration-response curves of L-glu on JumpIn-EAAT1 cells (±dox). **(E)** Combined concentration-inhibition curves of TFB-TBOA and UCPH-101 on +dox cells pretreated for 1 h with vehicle (PBS/DMSO) or increasing concentrations of TFB-TBOA or UCPH-101 and subsequently stimulated with a submaximal concentration (1 mM) of L-glu. **(F)** Combined concentration-response curves of L-glu on +dox cells pretreated for 1 h with vehicle (PBS/DMSO), 1 μM, 3.16 µM or 10 µM UCPH-101. **(G)** Combined concentration-response curves of L-glu on +dox cells pretreated for 1 h with vehicle (PBS/DMSO), 10 nM, 100 nM or 1 µM TFB-TBOA. Cellular response is expressed as the net AUC of the first 120 min after L-glu stimulation. Data are normalized to the response of 1 mM L-glu on +dox cells. Data are shown as the mean ± SEM of three to ten individual experiments each performed in duplicate.

Receptor-independent cellular responses by L-glu were assessed in non-transfected JumpIn-EAAT1 cells in the absence (−dox) or presence (+dox) of doxycycline. Incubation with doxycycline did not affect the growth of the cells prior to pretreatment or stimulation (Figure 2A). L-glu induced a gradual concentration-dependent increase in nCI in non-induced cells (pEC_50_ = 4.2 ± 0.3), whereas in dox-induced cells L-glu produced a drastically elevated nCI (pEC_50_ = 3.4 ± 0.0) reaching a 10-fold higher plateau after 120 min (Table 1, Figures 2B–D). Similar L-glu potencies were measured at 120 min in the TRACT assay on both non-induced and dox-induced JumpIn-EAAT1-mGluR_2_ and JumpIn-EAAT1-mock cells (Table 1). This indicates that the L-glu-induced cellular response is largely EAAT1-mediated. Unless stated otherwise, all further experiments were conducted on dox-induced cells.

To confirm the specific role of EAAT1 in the L-glu response we assessed the pharmacological properties of UCPH-101 and TFB-TBOA. No substantial changes in nCI were observed during the 1 h pretreatment with 10 µM of either inhibitor (Supplementary Figures S1E,F). Both UCPH-101 (pIC_50_ = 5.2 ± 0.1) and TFB-TBOA (pIC_50_ = 6.7 ± 0.1) blocked the response of 1 mM L-glu in a concentration-dependent manner (Table 1, Figure 2E). To assess the reproducibility and robustness of the inhibitory assay window, a semi-manual high-throughput screening (HTS) validation was performed using a low (10 µM TFB-TBOA), mid (0.2 µM TFB-TBOA), and high (vehicle) signal upon stimulation with 1 mM L-glu (Supplementary Figures S2A). The assay produced a Z’ of 0.85 ± 0.01 and a signal window (SW) of 25.1 ± 1.3, which indicates an “excellent” assay (Zhang et al., 1999; Iversen et al., 2004) that is suitable for single-point detection of EAAT1 inhibition (Supplementary Figures S2B).

The mechanism of inhibition by UCPH-101 and TFB-TBOA was demonstrated by pretreating cells with three different concentrations of inhibitor prior to stimulation with increasing concentrations of L-glu (Figures 2F,G). Since not all L-glu concentration-response curves reached a plateau within the tested concentration range, no formal Schild analysis was performed. Nevertheless, UCPH-101 reduced the maximum response of L-glu (Figure 2F) without much affecting the potency of L-glu, whereas the presence of TFB-TBOA produced an apparent right-ward shift of the L-glu curve (Figure 2G), indicating a non-competitive and competitive mode of inhibition, respectively.

### 3.3 Substrate-Specific Uptake *via* EAAT1 Induces Na^+^/K^+^-ATPase-Dependent Cell Spreading

**FIGURE 3:**
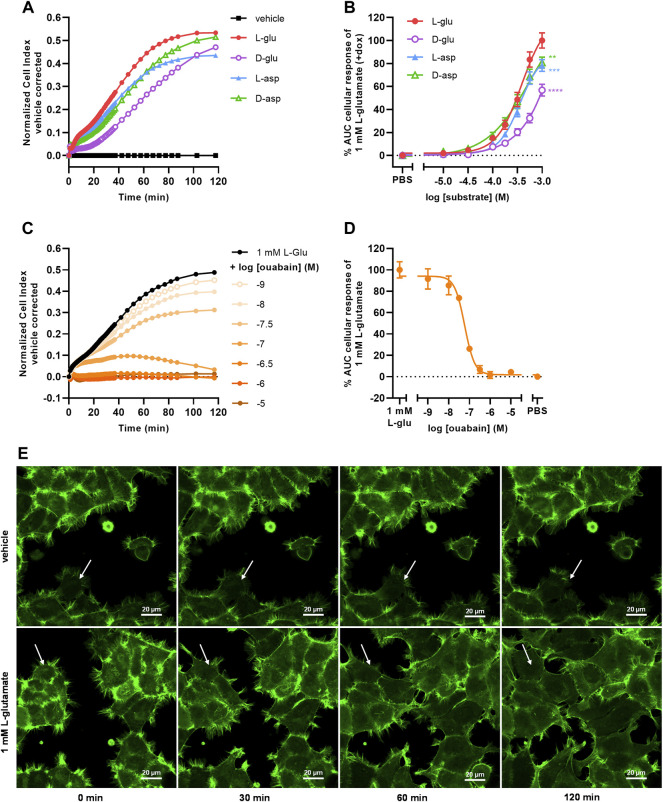
Substrate-dependent uptake *via* EAAT1 mediates Na^+^/K^+^-ATPase (NKA)-dependent cell spreading. **(A)** Vehicle-corrected nCI traces of +dox cells stimulated with vehicle (PBS), 1 mM of L-glutamate (L-glu), D-glutamate (D-glu), L-aspartate (L-asp) or D-aspartate (D-asp), shown as the mean of a representative experiment performed in duplicate. **(B)** Combined concentration-response curves of L-glu (derived from Figure 2D), D-glu, L-asp and D-asp on +dox cells. **(C)** Vehicle (PBS)-corrected nCI traces of +dox cells pretreated for 1 h with vehicle (PBS/DMSO, black) or increasing concentrations of ouabain and subsequently stimulated with 1 mM L-glu, shown as the mean of a representative experiment performed in duplicate. **(D)** Combined concentration-inhibition curves of ouabain in +dox cells. Vehicle (PBS)-induced responses were subtracted from L-glu-induced responses for each concentration of ouabain. Cellular response is expressed as the net AUC of the first 120 min after substrate stimulation. Data are normalized to the response of 1 mM L-glu. Data are shown as the mean ± SEM of three to six individual experiments each performed in duplicate. **(E)** Representative confocal images of JumpIn-EAAT1-LifeAct-GFP (green) cells 0, 30, 60, and 120 min after stimulation with vehicle (PBS) or 1 mM L-glu, scale bar = 20 µm. Stills were selected from a representative live imaging movie from two independent experiments each performed in triplicate. White arrow indicates representative behavior of a single cell, showing increased cell spreading over time.

To investigate whether the cellular responses were L-glu specific, three additional EAAT1 substrates were tested. Similar to L-glu, stimulation of cells with D-glutamate (D-glu), L-aspartate (L-asp), and D-aspartate (D-asp) induced a substantial nCI increase within 120 min (Figure 3A). L-asp and D-asp had potencies (pEC_50_ = 3.4 ± 0.0 and 3.4 ± 0.1, respectively) that were comparable to L-glu, whereas the potency of D-glu was substantially lower (pEC_50_ < 3.0) (Table 1, Figure 3B). The maximal responses of 1 mM L-asp (*p* = 0.0005), D-asp (*p* = 0.0054), and D-glu (*p* < 0.0001) were significantly lower than L-glu (Figure 3B), which was attributed to a lower nCI plateau (L-asp) or slower onset of the nCI increase (D-asp, D-glu) (Figure 3A). These data suggest that the cellular responses are substrate-specific and likely emanate from a shared mechanism.

To assess the role of Na^+^/K^+^-ATPase (NKA) in the L-glu-induced cellular response, cells were pretreated for 1 h with increasing concentrations of the NKA inhibitor ouabain and stimulated with 1 mM L-glu (Figure 3C). During pretreatment with ouabain the nCI gradually decreased in a concentration-dependent manner (Supplementary Figures S1G), which was corrected for during substrate stimulation by including vehicle controls for each concentration of ouabain. Upon L-glu stimulation ouabain showed concentration-dependent inhibition of the cellular response (pIC_50_ = 7.2 ± 0.0) (Table 1, Figure 3D). This validates that the Na^+^ gradient and NKA activity are crucial for proper EAAT1-mediated L-glu uptake and the subsequent cellular responses.

To determine whether changes in cell morphology underlie the L-glu-induced cellular responses, JumpIn-EAAT1 cells were transduced with a LifeAct-GFP actin lentiviral plasmid (JumpIn-EAAT1-LifeAct-GFP) and visualized during an L-glu stimulation using confocal microscopy (Figure 3E). Dox-induced cells did not show substantial changes in morphology within 2 h after vehicle stimulation. Strikingly, cells stimulated with 1 mM L-glu appeared to drastically extend their actin cytoskeleton outward within 2 h after stimulation, producing protrusions that stretch towards neighboring cells effectively expanding their surface area and thus well coverage (Figure 3E, Supplementary Movies S5A,B). When cells were pretreated with 1 µM ouabain, stimulation with vehicle or 1 mM L-glu did not induce cell spreading, but rather a slight reduction of the cell surface area (Supplementary Figures S3, Supplementary Movies S5C,D). Since the onset of cell spreading concurs with the nCI increases that were observed in xCELLigence experiments (Figure 2C), this implies that these distinct L-glu-induced morphological changes are at the basis of EAAT1-mediated cellular responses.

### 3.4 EAAT1-Mediated Uptake of L-Glu and L-Asp Affect Intracellular Metabolite Levels

**FIGURE 4:**
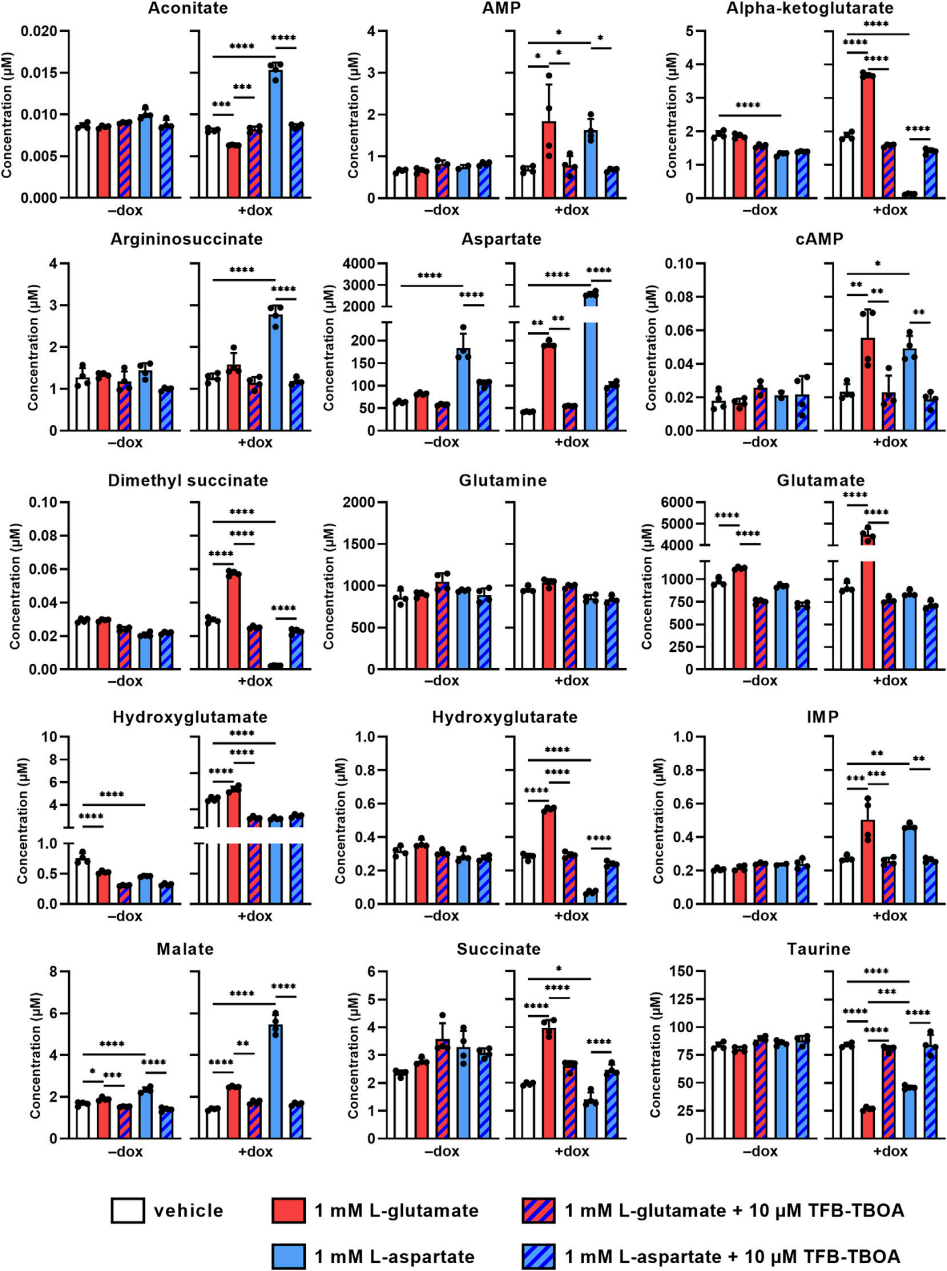
Changes in intracellular metabolite levels upon substrate stimulation of JumpIn-EAAT1 cells. Targeted metabolomics was used to measure the concentrations of several metabolites in the absence (−dox) or presence (+dox) of doxycycline. AMP, adenosine monophosphate; cAMP, cyclic AMP; IMP, inosine monophosphate. Cells were pretreated for 1 h with vehicle (PBS/DMSO, plain bars) or 10 µM TFB-TBOA (hatched bars) prior to stimulation with vehicle (PBS, white bars), 1 mM L-glutamate (red bars) or L-aspartate (blue bars). Data are shown as the mean concentration (in µM) ± SD of four replicate experiments. *p < 0.05, **p < 0.01, ***p < 0.001, ****p < 0.0001; one-way ANOVA with Tukey’s post-hoc test.

Since glutamate and aspartate may be metabolized in the cell upon entering the cytosol *via* EAAT1, we investigated whether changes in intracellular metabolite levels could provide an explanation for the observed cellular responses. Non-induced and dox-induced cells were stimulated for 2 h with 1 mM L-glu or L-asp in the presence or absence of 10 µM TFB-TBOA and lysates were subsequently analyzed using targeted metabolomics. Intracellular levels of 131 predefined metabolites were screened and visualized in volcano plots to highlight significant increases or decreases after 2 h (Supplementary Figure S4, Supplementary Table S6). Based on these plots, we focused on intracellular metabolites that were significantly increased or decreased for both L-glu and L-asp in order to reveal a common mechanism (Figure 4). Firstly, metabolite levels were generally not affected in non-induced (−dox) cells. Secondly, in dox-induced (+dox) cells, stimulation with L-glu and L-asp resulted in a respective 5-fold and a 60-fold increases of glutamate and aspartate, respectively, which confirms that both substrates entered the cells *via* EAAT1. Thirdly, all observed changes in metabolite levels (except hydroxyglutamate) were significantly prevented in the presence of TFB-TBOA (Figure 4, hatched bars), indicating that these were the result of EAAT1-mediated substrate influx.

The levels of glutamine, a product of glutamate metabolism, were not significantly altered by either substrate, indicating that this metabolite is not involved in the cellular response. Interestingly, the tricyclic acid (TCA) cycle intermediates (alpha-ketoglutarate, dimethyl succinate, hydroxyglutamate, hydroxyglutarate, succinate and aconitate) were oppositely increased or decreased for each substrate, which suggests that L-glu and L-asp are differentially metabolized in the cell and that the TCA cycle does not contribute to the cellular response. Moreover, products of L-asp metabolism [argininosuccinate, adenosine monophosphate (AMP), inosine monophosphate (IMP), and malate] were increased upon stimulation with L-asp, but to a lesser extent by L-glu. Strikingly, the organic osmolyte taurine, which is not a product of L-glu or L-asp metabolism and is involved in cell volume regulation, was significantly reduced upon stimulation with both L-glu and L-asp, which suggests that substrate uptake *via* EAAT1 may result in taurine efflux. Taken together, these data imply that the cellular response in the impedance-based assay is likely not driven by substrate metabolism, but is rather associated with changes in cell volume.

### 3.5 MDA-MB-468 Cells With Endogenous EAAT1 Expression Show Substrate-Induced Cellular Responses

**FIGURE 5:**
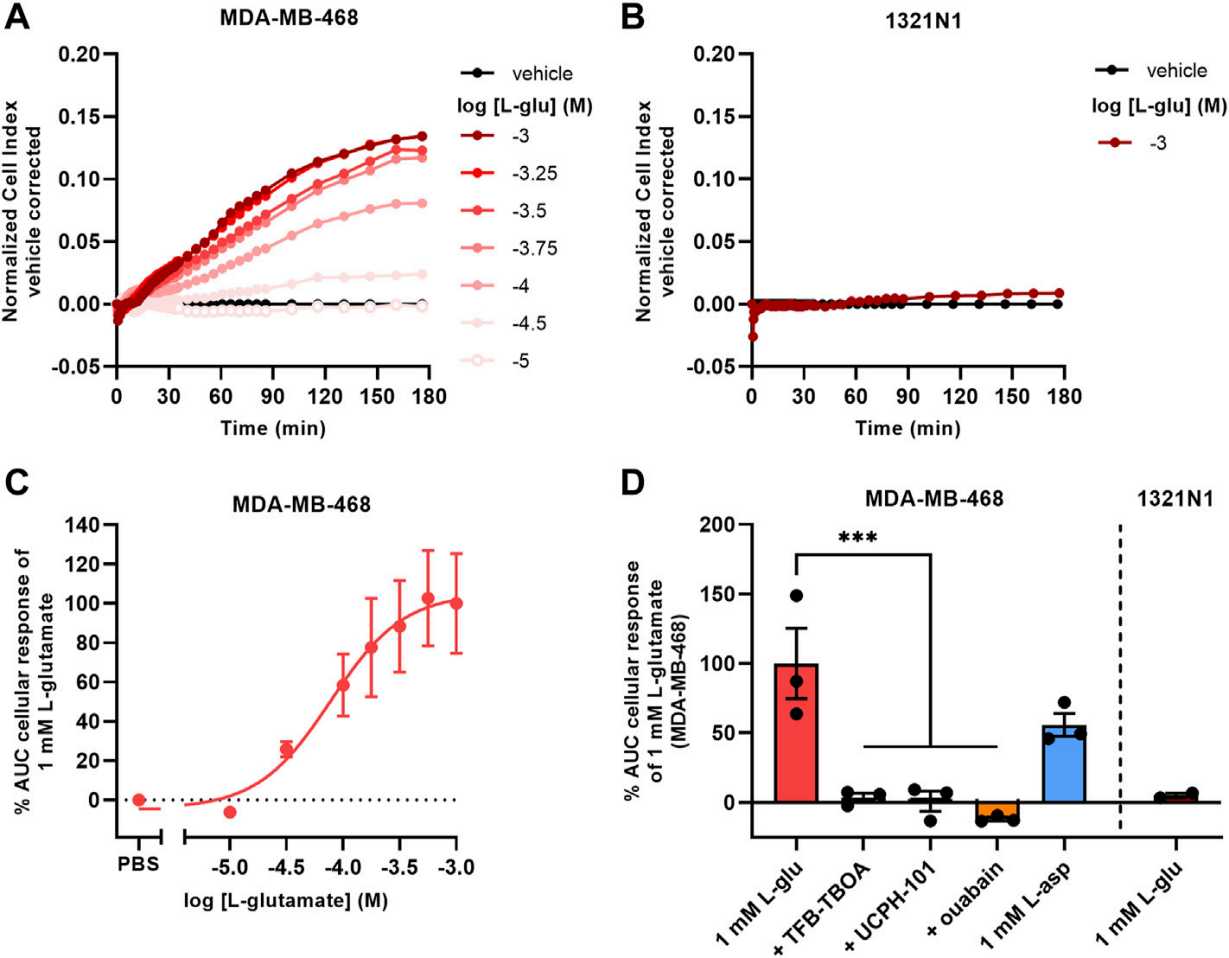
L-glutamate induces cellular responses on MDA-MB-468 cells with endogenous EAAT1 expression. **(A,B)** Vehicle-corrected nCI traces of MDA-MB-468 **(A)** and 1321N1 **(B)** cells stimulated with L-glutamate (L-glu), shown as the mean of a representative experiment performed in duplicate. **(C)** Combined concentration-response curve of L-glu on MDA-MB-468 cells. **(D)** Cellular response of L-glu or L-aspartate (L-asp) on MDA-MB-468 and 1321N1 cells. MDA-MB-468 cells were pretreated for 1 h with vehicle (PBS/DMSO), 10 µM TFB-TBOA, 10 µM UCPH-101 or 1 µM ouabain prior to stimulation with vehicle (PBS), 1 mM L-glu or L-asp. Data are shown as the mean ± SEM of three (MDA-MB-468) or SD of two (1321N1) individual experiments each performed in duplicate. ***p < 0.001; one-way ANOVA with Dunnett’s post-hoc test.

To assess whether EAAT1-mediated cellular responses were not exclusive to JumpIn-EAAT1 cells, we selected two cell lines with high or low endogenous EAAT1 expression. Transcriptomics analyses indicated that the human breast cancer cell line MDA-MB-468 has a high endogenous expression of EAAT1 (BioSamples database: SAMN11893674, SAMN11893681, SAMN11893688), whereas the human astrocytoma cell line (1321N1) lacked endogenous EAAT1 expression (BioSamples database: SAMN11893671, SAMN11893678, SAMN11893685). In line with the expression data, MDA-MB-468 cells showed a gradual and concentration-dependent nCI increase upon stimulation with L-glu (pEC_50_ = 4.0 ± 0.2) that reached a plateau after 180 min (Figures 5A,C). In contrast, 1321N1 cells did not show a substantial cellular response upon stimulation with 1 mM L-glu within 180 min (Figures 5B,D). The response of 1 mM L-glu on MDA-MB-468 cells was completely blocked in the presence of 10 µM TFB-TBOA and 10 µM UCPH-101, which suggested that the cellular response was EAAT1-mediated (Figure 5D). Similar to JumpIn-EAAT1 cells the cellular responses were substrate- and Na^+^-dependent, which was demonstrated by an L-asp response and inhibition of the L-glu response by ouabain, respectively. These data suggest that EAAT1 function can be assessed in cells with endogenous EAAT1 expression.

### 3.6 L-Glutamate Induces Cellular Responses *via* EAAT2 and EAAT3, but not EAAT4, and EAAT5

**FIGURE 6:**
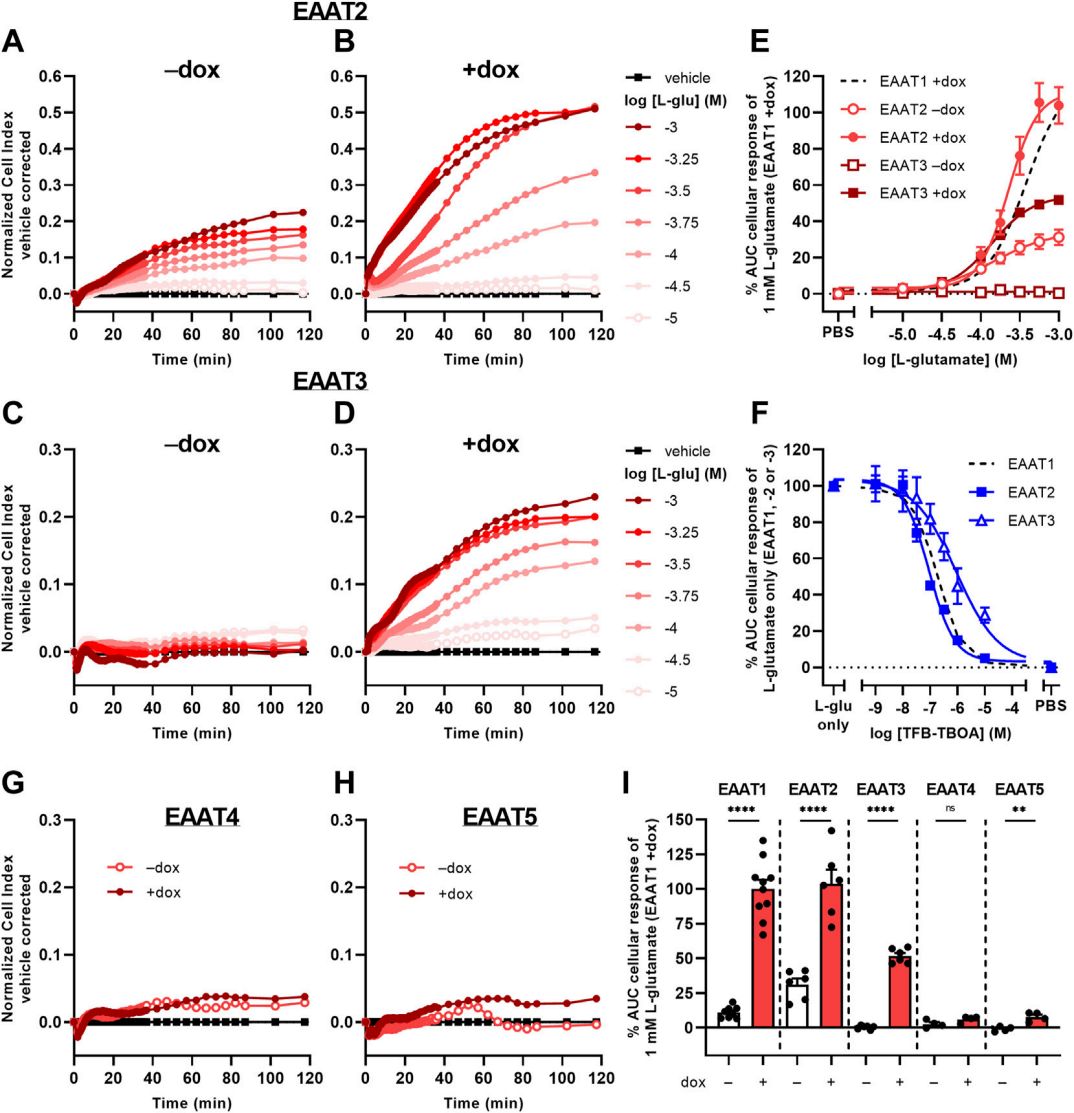
L-glutamate-induced cellular responses on JumpIn-EAAT2 and JumpIn-EAAT3 cells. **(A**–**D**,**G**,**H)** Vehicle-corrected nCI traces of JumpIn-EAAT2 **(A**,**B)**, JumpIn-EAAT3 **(C**,**D)**, JumpIn-EAAT4 **(G)** and JumpIn-EAAT5 **(H)** cells in the absence (−dox) or presence (+dox) of doxycycline stimulated with vehicle (PBS) or L-glutamate (L-glu), shown as the mean of a representative experiment performed in duplicate. **(E)** Combined concentration-response curves of L-glu on JumpIn-EAAT2 and JumpIn-EAAT3 cells (±dox). Data are normalized to the response of 1 mM L-glu on JumpIn-EAAT1 +dox cells (data is derived from Figure 2D and shown as a black dotted line). **(F)** Combined concentration-inhibition curves of TFB-TBOA on +dox JumpIn-EAAT2 and JumpIn-EAAT3 cells pretreated for 1 h with vehicle (PBS/DMSO) or increasing concentrations of TFB-TBOA and subsequently stimulated with a submaximal concentration (316 µM) of L-glu. Data are normalized to the response of 1 mM L-glu (JumpIn-EAAT1, data is derived from Figure 2E and shown as a black dotted line) or 316 μM L-glu (JumpIn-EAAT2 and -EAAT3). **(I)** Cellular response of 1 mM L-glu on−dox and +dox JumpIn cells for all EAAT subtypes. Data on JumpIn-EAAT1, -EAAT2 and -EAAT3 cells were derived from Figure 2D and Panel 6E. Cellular response is expressed as the net AUC of the first 120 min after substrate stimulation. Data are shown as the mean ± SEM of at least three individual experiments each performed in duplicate. ns = not significant, **p < 0.01, ****p < 0.0001; unpaired two-tailed Student’s t-test.

To investigate whether the phenotypic assay could be used to assess activity of other glutamate transporters, we used JumpIn cell lines with dox-inducible overexpression of EAAT2, EAAT3, EAAT4, and EAAT5 (Figure 6). Non-induced (−dox) JumpIn-EAAT2 cells responded to L-glu stimulation in a concentration-dependent manner (pEC_50_ = 3.8 ± 0.1), whereas no substantial L-glu response was observed in non-induced JumpIn-EAAT3 cells (Figures 6A,C, Table 1). Similar to JumpIn-EAAT1 cells, L-glu induced concentration-dependent nCI increases reaching a plateau after 120 min on dox-induced (+dox) JumpIn-EAAT2 (pEC_50_ = 3.6 ± 0.0) and JumpIn-EAAT3 cells (pEC_50_ = 3.9 ± 0.0) (Figures 6B,D,E, Table 1). The responses of a submaximal concentration (316 µM) of L-glu could be inhibited in a concentration-dependent manner by TFB-TBOA in JumpIn-EAAT2 (pIC_50_ = 7.1 ± 0.0) and JumpIn-EAAT3 (pIC_50_ = 6.1 ± 0.2) cells, which indicates that the cellular responses were transporter-specific (Figure 6F).

The L-glu-induced responses in dox-induced JumpIn-EAAT4 (Figure 6G) and JumpIn-EAAT5 cells (Figure 6H) were substantially lower than for cells overexpressing EAAT1, EAAT2 or EAAT3 (Figure 6I). L-glu induced a significantly higher response in dox-induced JumpIn-EAAT5 cells than in non-induced cells (*p* = 0.0032), whereas in JumpIn-EAAT4 this difference was not significant (*p* = 0.093). Immunoblotting of JumpIn-EAAT cell lysates indicates that the relative expression of EAAT4 and EAAT5 was considerably lower than EAAT1, EAAT2, and EAAT3 (Supplementary Figure S7), which indicates that the transporter expression level could be related to the magnitude of the cellular response. Taken together, these data confirm that the phenotypic impedance-based assay allows pharmacological assessment of at least three Na^+^-dependent glutamate transporters.

## 4 Discussion

In recent years the pleas for invigoration of fundamental SLC drug research have led to an ever-growing wealth of innovative molecular tools and technologies that enable functional investigation of transport proteins (César-Razquin et al., 2015; Superti-Furga et al., 2020; Dvorak et al., 2021). In efforts to expand the SLC toolbox, we demonstrate a novel method to detect activity of glutamate transporters in living cells without the use of labels or biochemical reporters. Impedance-based biosensors can detect in real-time the temporal cytoskeleton rearrangements that result from GPCR activation in live cells (Hillger et al., 2017; Doijen et al., 2019), making this an ideal functional readout. As such, we initially attempted to set up a TRACT assay, as previously reported by our lab (Vlachodimou et al., 2019; Sijben et al., 2021a, 2021b), for EAAT1 by using a cell line with inducible overexpression of EAAT1 and transient expression of mGluR_2_. However, in the absence of mGluR_2_, we observed that JumpIn-EAAT1 cells start spreading as a result of EAAT1-mediated L-glu uptake (Figure 3E), which was reflected by the increased cellular response in the impedance assay (Figure 2). This phenotypic response suggests a very different mechanism by which transport activity of EAAT1 can be assessed.

The interpretation of cellular impedance data requires an initial mechanistic understanding of the subcellular events that underlie the major changes in cell morphology as a response to applied external stimuli (Kho et al., 2015). Since the cellular response in JumpIn-EAAT1 cells did not arise from GPCR activation in the absence of mGluR_2_, we sought to investigate the possible triggers that induced cell spreading. Initially, we hypothesized that increased intracellular levels of L-glu may lead to conversion of L-glu to glutamine or tricarboxylic acid (TCA) cycle intermediates, which could possibly be associated with ATP synthesis and changes in cell morphology (Magi et al., 2013). However, in line with initial pharmacological characterizations of EAAT1 (Arriza et al., 1994; Jensen and Bräuner-Osborne, 2004), both L- and D-isomers of glutamate and aspartate induced cellular responses of comparable magnitude (Figures 3A,B). Since these substrates have divergent metabolic fates, this suggests that it is unlikely that the cellular response is the result of intracellular substrate metabolism. Indeed, upon stimulation with L-glu or L-asp we observed opposite changes in the levels of the tricarboxylic acid (TCA) cycle intermediates and related metabolites, which either increased (L-glu) or decreased (L-asp) (Figure 4). This suggests that L-glu and L-asp differentially feed into the TCA cycle (Andersen et al., 2021) and as such it is unlikely that this metabolic route underlies the observed phenotypic response of both substrates. Interestingly, intracellular aspartate was increased 60-fold upon stimulation with L-asp, but only 5-fold with L-glu. Pathways that are associated with L-asp metabolism, such as purine nucleotide synthesis (AMP, IMP), the urea cycle (AMP, argininosuccinate) and the malate-aspartate shuttle (malate) also showed increased metabolite levels (Arinze, 2005). However, since L-asp induces a slightly lower cellular response in the impedance-based assay compared to L-glu, it is unlikely that these changes in L-asp metabolite levels explain the changes in cell morphology. It should be noted that some metabolite levels (e.g., hydroxyglutamate) could have been altered as a consequence of the presence of doxycycline rather than the induced expression of the transporter, for these types of antibiotics have been reported to induce mitochondrial stress responses (Moullan et al., 2015). Nevertheless, any non-specific metabolite changes upon L-glu or L-asp stimulation were identified by the absence of substrate-specific regulation and/or a lack of inhibition by TFB-TBOA. Although metabolomics provides a wealth of information on the intracellular fates of transporter-mediated substrate uptake, we conclude that substrate metabolism is not the main driver behind the substrate-induced cellular responses.

As such, we ascribed the cellular responses to the initial changes in intracellular ionic concentrations and cell volume following EAAT-mediated substrate uptake. The continuous Na^+^-driven uptake of substrate *via* EAAT leads to an accumulation of intracellular Na^+^ and substrate with release of 1 K^+^ with each transport cycle. The Na^+^ gradient-restoring activity of NKA leads to subsequent elevation of intracellular K^+^ (Rose et al., 2009). The net increase of the intracellular positive charges with each transport cycle is compensated by the influx of Cl^−^ *via* the uncoupled anion conductance of EAAT or other Cl^−^-coupled transporters (Grewer and Rauen, 2005), which leads to an increased osmolarity that evokes water flux into the cytosol and causes cell swelling (Hoffmann et al., 2009; Rungta et al., 2015). The resulting cell volume increase causes the formation of membrane protrusions that extend towards regions with high extracellular substrate concentrations (Figure 3E), commencing a transmembrane ionic cycle that drives cell swelling and spreading at the leading edges of the cell membrane similar to a migrating cell (Schwab et al., 2006; Morishita et al., 2019). It should be noted that we did not measure the actual swelling of the cells in this study, and as such the specific contribution of cell swelling to the observed cellular responses remains to be experimentally verified. Nevertheless, our hypothesis is in agreement with previous reports of glutamate- and aspartate-induced cell swelling in astrocytes (Chan et al., 1990; Schneider et al., 1992; Staub et al., 1993; Bender et al., 1998; Hansson et al., 2000; Koyama et al., 2000; Wilson and Mongin, 2018), which have related swelling to EAAT-mediated cytosolic accumulation of Na^+^, K^+^, and Cl^−^ ions. In line with this, inhibition of NKA by ouabain disrupts the Na^+^ gradient across the membrane and prevents substrate uptake and cellular responses *via* EAAT (Figure 3D). However, reduced NKA activity does not block, but may rather enhance the Cl^−^ conductance of EAAT (Kovermann et al., 2022). Thus, it is likely that the entry of large quantities of Na^+^ *via* EAAT upon substrate stimulation, and not primarily the uncoupled Cl^−^ flux, is the major trigger for cell volume changes and subsequent cell spreading.

**FIGURE 7:**
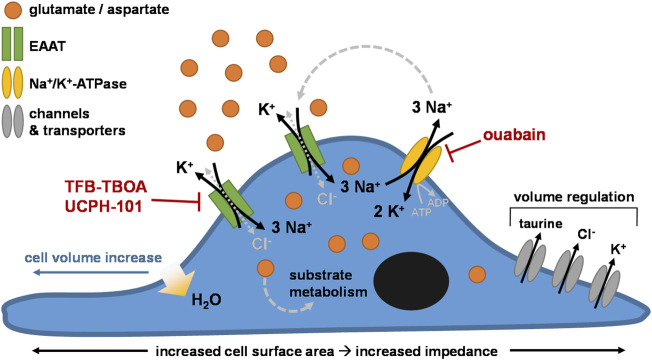
Proposed mechanism of the phenotypic impedance-based assay. Cells (over)expressing excitatory amino acid transporters (e.g., EAAT1) are stimulated with exogenous substrate (e.g., glutamate). The substrate is taken up into the cell through EAAT together with 3 Na^+^ in exchange for 1 K^+^. Cl^−^ enters the cells *via* uncoupled Cl^−^-conductivity of EAAT or other Cl^−^ influx mechanisms, counterbalancing the increase in intracellular Na^+^. The Na^+^/K^+^-ATPase (NKA) restores the transmembrane Na^+^ gradient by ATP-dependent efflux of 3 Na^+^ in exchange for 2 K^+^. Over time, the intracellular concentrations of substrate, Na^+^, K^+^, and Cl^−^ ions initially rise, increasing cell osmolarity. Subsequent cell swelling, *via* influx of H_2_O into the cytosol, triggers cell spreading, which increases the surface area of the E-plate that is covered by cells. Regulatory mechanisms lead to dissipation of the high intracellular ion concentrations *via* channels and/or transporters that mediate efflux of Cl^−^, K^+^, and osmolytes such as taurine, effectively reducing cell volume over time.

To compensate for increased osmolarity, high intracellular ion levels are eventually dissipated *via* the release of K^+^ and Cl^−^ as well as organic osmolytes through volume-sensitive channels (Rungta et al., 2015; Schober and Mongin, 2015). Indeed, in L-glu- and L-asp-stimulated JumpIn-EAAT1 cells we observed a significant decrease of the intracellular concentration of taurine (Figure 4), which is an osmolyte that is released in response to cell swelling to decrease cell volume (Hoffmann et al., 2009; Lambert et al., 2015). In addition to increased osmolarity, cell swelling is accompanied by increased cytosolic Ca^2+^ concentrations, which together with mechanical cell stress evoke the release of adenosine triphosphate (ATP) from the cell (Okada et al., 2001; Boudreault and Grygorczyk, 2004). Autocrine activation of metabotropic P2Y purinergic receptors by ATP can in turn lead to increased cytosolic cyclic AMP (cAMP) levels, further Ca^2+^ elevations, activation of Ca^2+^-dependent K^+^ and Cl^−^ channels, cytoskeleton remodeling and increased efflux of Cl^−^ and taurine *via* volume-regulated anion channels (VRAC) (Jakab et al., 2002; Franco et al., 2008; Jentsch, 2016; Wilson and Mongin, 2018). Of note, we observed significant elevations of cAMP levels in JumpIn-EAAT1 cells upon stimulation with L-glu and L-asp (Figure 4), which suggests that GPCR activation is involved in the cellular response (Franco et al., 2008). In general, reorganization of the actin cytoskeleton is a common consequence of cell volume regulation and the mechanisms driving these events have been extensively described in literature (Lang et al., 1998; Okada et al., 2001; Jakab et al., 2002; Jakab and Ritter, 2006; Lang, 2007; Hoffmann et al., 2009). Based on our observations, we conclude that the phenotypic responses in the impedance assay are likely initiated by EAAT-mediated cell swelling (Figure 7).

The phenotypic assay was used to assess functionality of various EAAT subtypes in overexpressing cells, as well as EAAT1 function in cells endogenously expressing the transport protein. Interestingly, the potencies of L-glu for EAAT1-3 that we found in the phenotypic assay were substantially lower than the reported steady-state affinities (K_m_) of L-glu (around 20 µM) for these transporters (Arriza et al., 1994; Tzingounis and Wadiche, 2007). It should be noted that the impedance-based method does not provide information on uptake kinetics, as it is not a direct measure of substrate uptake and as such the potencies found here should be compared to literature with caution. Nevertheless, a high transporter density has been shown to affect the buffering capacity of the substrate, resulting in a reduced affinity for the substrate (Sun et al., 2014). Indeed, dox-induced JumpIn-EAAT1 cells had a lower L-glu potency than MDA-MB-468 cells with endogenous EAAT1 expression, likely due to a higher transporter density in the former cell line. Of note, a higher L-glu potency was observed in non-induced JumpIn-EAAT cells, which could indicate low levels of endogenous EAAT or “leaky” heterologous transporter expression in the absence of dox (Costello et al., 2019), although this was not apparent from Western blots of −dox cell lysates (Supplementary Figure S7). Moreover, the differences in L-glu potency between EAAT1-3 could be attributed to their dissimilar substrate turnover rate, which is 2- to 10-fold higher for EAAT3 than EAAT2 and EAAT1, respectively (Vandenberg and Ryan, 2013). Besides discrepancies in L-glu potency, we also observed a difference in the maximal response of L-glu between the various EAAT subtypes (Figure 6). A likely explanation for the lack of a specific L-glu response for EAAT4 and EAAT5 is the poor expression of these subtypes compared to EAAT1-3, although a small fraction of these transporters appears to be expressed (Supplementary Figure S7). Alternatively, the absence of a substantial substrate responses for EAAT4 and EAAT5 may be related to slow turnover rates (Vandenberg and Ryan, 2013) and their predominant chloride conductivity function (Fairman et al., 1995; Lukasiewcz et al., 2021; Ryan et al., 2021).

One of the applications of the phenotypic assay is to screen for potential EAAT modulators. We have validated the EAAT specificity of the substrate-induced cellular response by using TFB-TBOA and UPCH-101, which are widely used tool compounds for EAATs (Shimamoto et al., 2004; Jensen et al., 2009). Strikingly, the inhibitory potency values that were found in the cell swelling assay were roughly 10-fold lower than in radioligand uptake assays (Shimamoto et al., 2004; Jensen et al., 2009; Abrahamsen et al., 2013). Although these assays use different readouts, one explanation for this discrepancy might be the high competing concentrations of substrate in the cell swelling assay, which are 100–10,000 times higher than in radioligand uptake assays (Fontana, 2018). Alternatively, dox-induced overexpression of EAATs could create a pool of “spare” transporters on the plasma membrane that effectively buffers the extracellular concentration of inhibitor, resulting in a rightward shift of the concentration-inhibition curve and a lower apparent inhibitory potency (Belo do Nascimento et al., 2021). Although the transporter expression levels and substrate concentrations should be thoroughly considered in further assay development, our impedance-based assay showed a robust window for the detection of transporter inhibitors.

In summary, this study presents a novel application of a label-free biosensor to study function and pharmacology of a transport protein family. It provides an alternative to existing radioactive- or fluorescence-based methods and opens up new venues to study other Na^+^-coupled transporters or, in fact, any transporter of which the activity is intrinsically linked to cytoskeletal changes upon perturbation. In addition, we demonstrated that this method is sensitive enough to detect EAAT1 activity in an endogenous cell line, which could further expand the possibilities to investigate disease-relevant cell lines. Moreover, a semi-manual HTS validation demonstrates an “excellent” assay window (Zhang et al., 1999), which renders this phenotypic assay applicable for drug discovery screens. Ultimately, this methods is a novel addition to the continuously expanding drug discovery toolbox for SLC transporters.

## Data Availability

The datasets presented in this study can be found in online repositories. The names of the repository/repositories and accession number(s) can be found below: Transcriptomics analyses of MDA-MB-468 and 1321N1 cell lines are available in the BioSamples database (http://www.ebi.ac.uk/biosamples) under accession numbers SAMN11893671, SAMN11893678, SAMN11893685, SAMN11893674, SAMN11893681, and SAMN11893688. The targeted metabolomics dataset that was used for this study will be deposited in the MetaboLights repository (https://www.ebi.ac.uk/metabolights/). All other data presented in this study are included in the article and Supplementary Material.
