# Supplementary material for: Impedance-Based Phenotypic Readout of Transporter Function: A Case for Glutamate Transporters
Source: Front Pharmacol. 2022 May 23;13:872335. doi: 10.3389/fphar.2022.872335 (PMC9169222; doi:10.3389/fphar.2022.872335)
Supplement: Supplementary file 3 [file DataSheet1.DOCX]

Supplementary Material


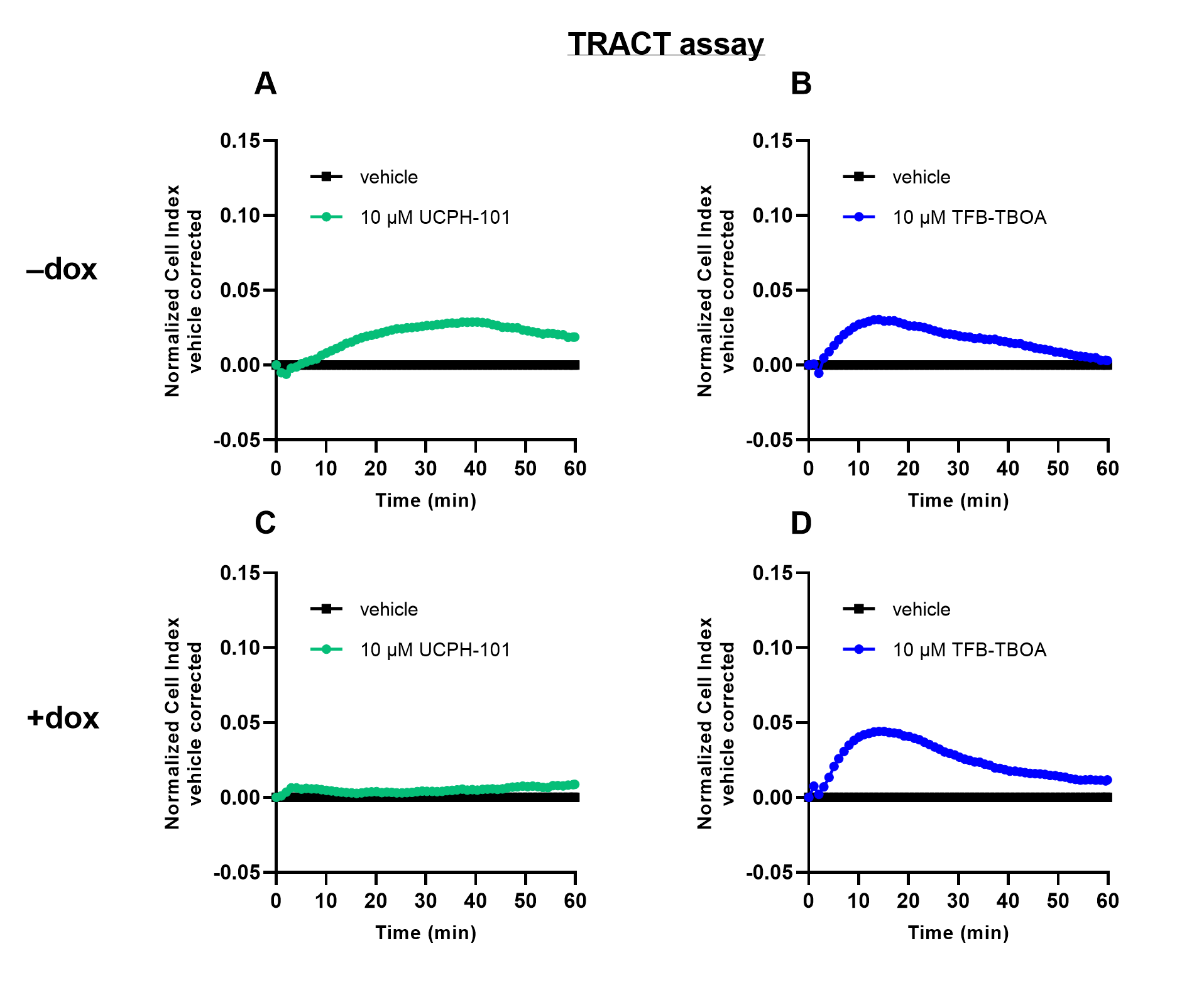

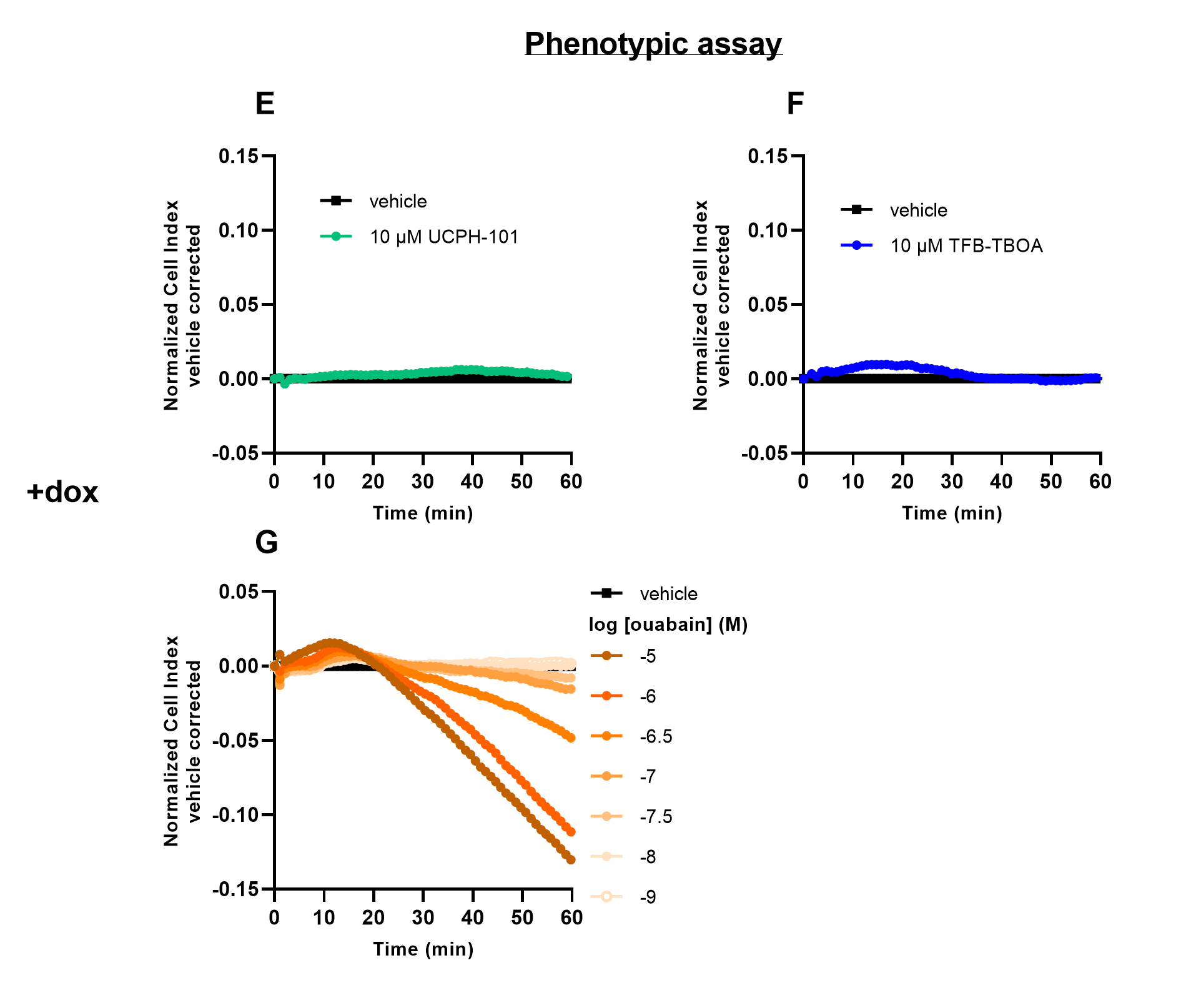


**Supplementary Figure 1.** Vehicle-corrected nCI traces during pretreatment of cells. (**A-D**) In the TRACT assay, JumpIn-EAAT1-mGluR_2_ cells were pretreated for 1 h with vehicle (PBS/DMSO), 10 µM UCPH-101 (**A**) or 10 µM TFB-TBOA (**B**) in the absence (–dox) (**A,B**) or presence (+dox) (**C,D**) of doxycycline. (**E,G**) In the phenotypic assay, JumpIn-EAAT1 +dox cells were pretreated for 1 h with vehicle (PBS/DMSO), 10 µM UCPH-101 (**E**), 10 µM TFB-TBOA (**F**) or increasing concentrations of ouabain (**G**). Data are shown as the mean of a representative experiment performed with four or eight replicates.


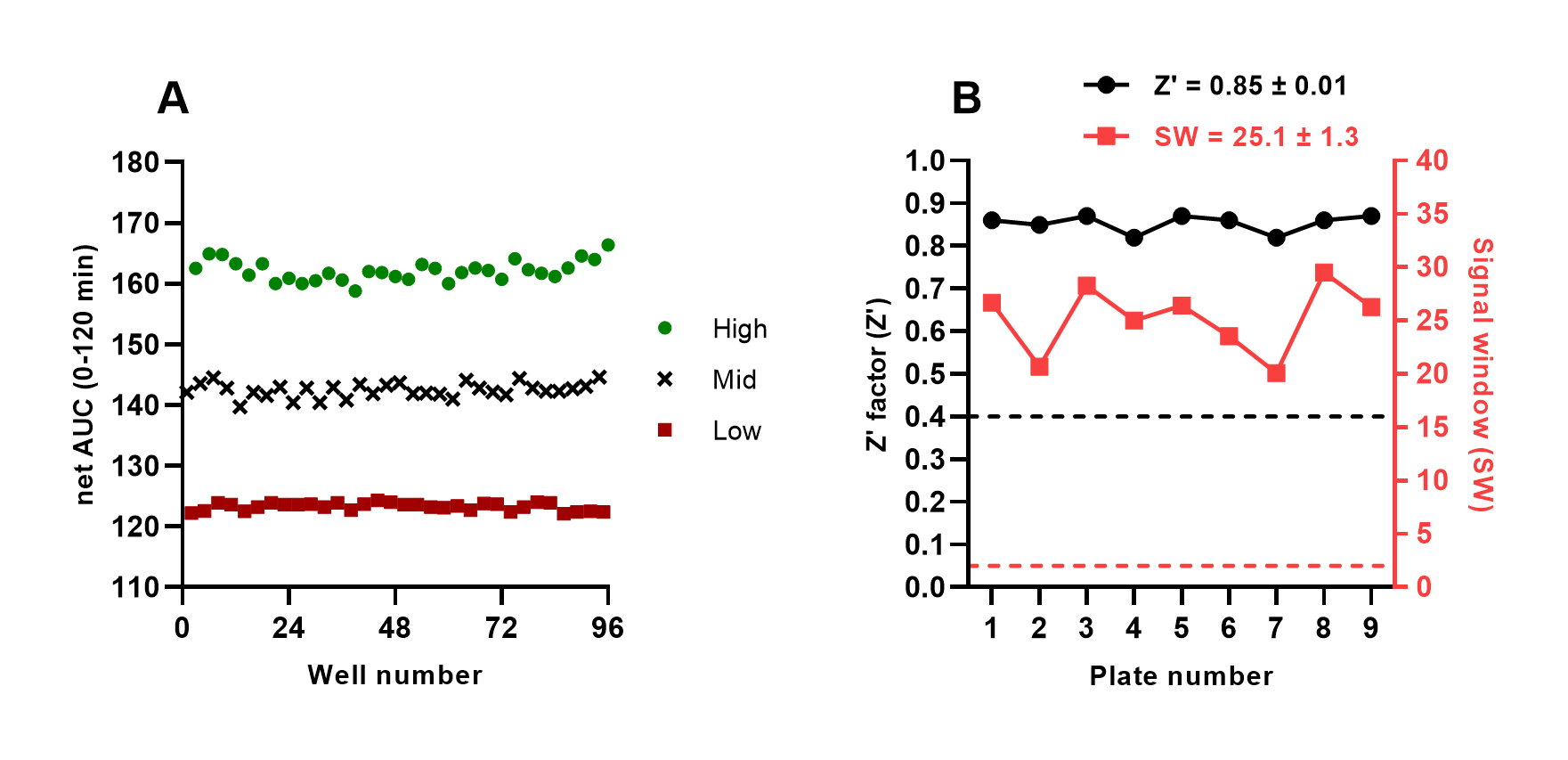


**Supplementary Figure 2.** HTS validation of the phenotypic impedance-based assay on JumpIn-EAAT1 cells. (**A**) Representative row-oriented graph of a 96-well E-plate contain high (vehicle + 1 mM L-glu), mid (0.2 µM TFB-TBOA + 1 mM L-glu) and low (10 µM TFB-TBOA + 1 mM L-glu) signal wells. Data is shown as the net AUC of the non-corrected nCI traces of the first 120 min after L-glu stimulation. Each data point represents a single well. (**B**) Stability of the Z’ factor (Z’) and signal window (SW) for each of the 96-well E-plates in the HTS validation. Three plates were run consecutively per day for three days in a row. Z’ and SW are calculated according to the formulas in the STAR methods and presented as the mean ± SEM of all nine plates.


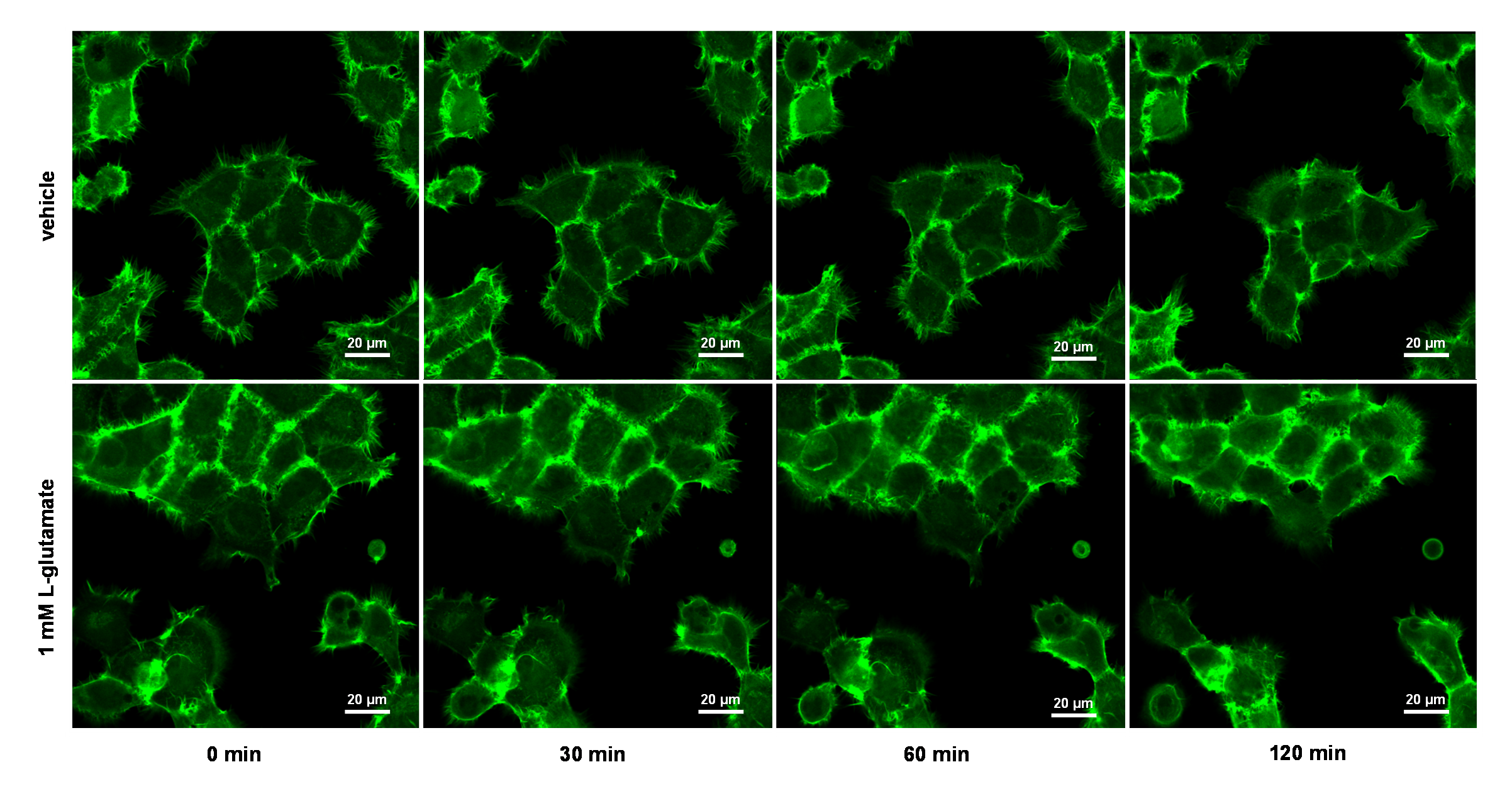


**Supplementary Figure 3.** NKA inhibition with ouabain does not increase L-glutamate-induced cell spreading. Representative confocal images of JumpIn-EAAT1-LifeAct-GFP (green) cells pretreated for 60 min with 1 µM ouabain. Images were taken 0, 30, 60 and 120 min after stimulation with vehicle (PBS) or 1 mM L-glu, scale bar = 20 µm. Stills were selected from a representative live imaging movie from two independent experiments each performed in triplicate.

**Supplementary Figure 4.** Volcano plots showing the log_2_ fold change of 131 metabolites plotted against the –log_10_ adjusted p-value. The change in metabolite levels is calculated for (**A,B**) non-induced (–dox) and (**C,D**) dox-induced (+dox) JumpIn-EAAT1 cells as the difference between vehicle treated cells and (**A,C**) L-glutamate or (**B,D**) L-aspartate treated cells. In addition, a comparison is made between (**E,F**) non-induced and dox-induced cells stimulated with (**e**) L-glutamate or f) L-aspartate. Changes in metabolite levels were considered substantial and significant when the log_2_ fold change ≤ -0.5 or ≥ 0.5, and the p-value < 0.05 (–log_10_ > 1.3).


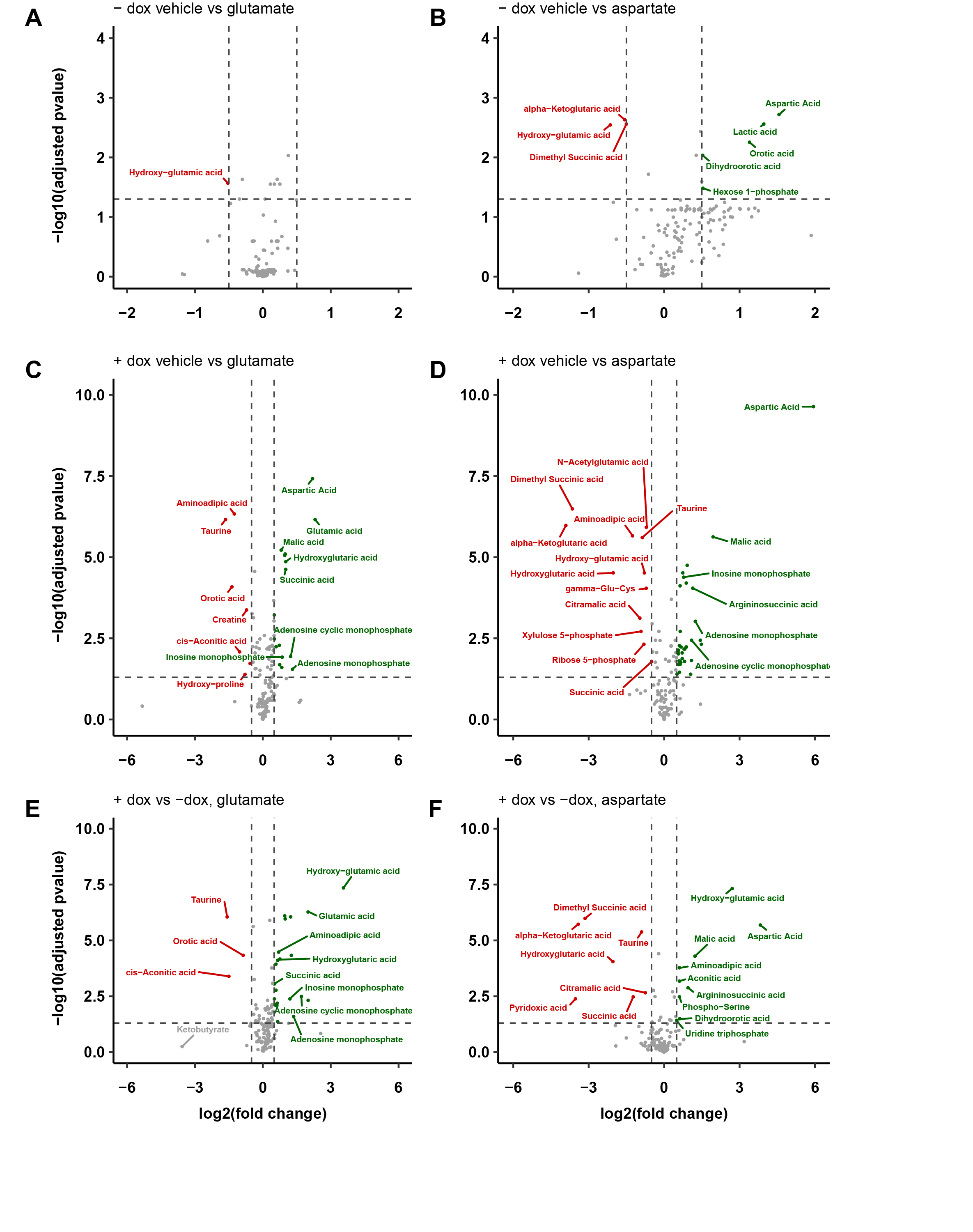


**Supplementary Movie 5.** Live cell imaging of JumpIn-EAAT1-LifeAct-GFP cells. Videos depict dox-induced cells pretreated with (**A,B**) vehicle (PBS/DMSO) or (**C,D**) 1 µM ouabain within the first 2 h after stimulation with (**A,C**) vehicle (PBS) or (**B,D**) 1 mM L-glutamate. Images were acquired every 5 minutes. Stills from these videos are shown in **Figure 3A** and **Supplementary Figure 3**.

**Supplementary Table 6.** Raw metabolomics data. Table indicates the condition (vehicle, TFB-TBOA pretreatment, L-glutamate or L-aspartate simulation), replicate number, dox-treatment, metabolite and concentration in µM.


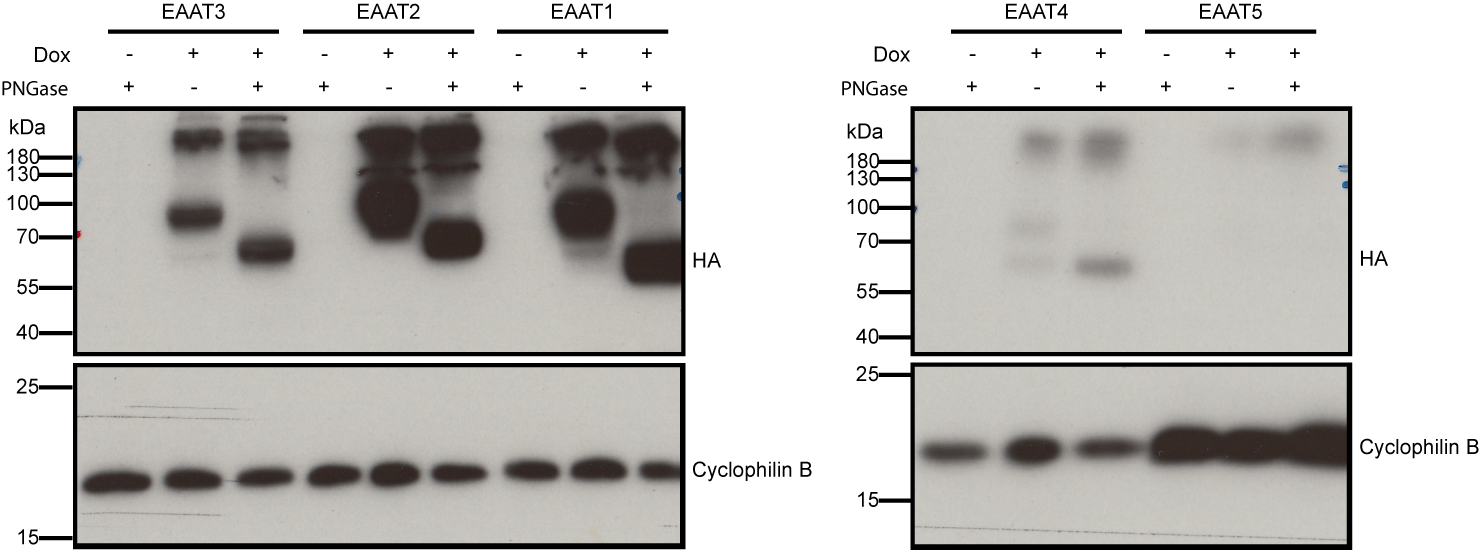


**Supplementary Figure 7.** Western blots of lysed JumpIn cells expressing hemagglutinin (HA)-tagged EAAT1-5. Cells were incubated for 24 h in the presence or absence of 1 µg/ml doxycycline (dox) prior to cell lysis. Where indicated, cell lysates were treated with PNGase to deglycosylate the protein. The expected molecular weights of deglycosylated protein are: EAAT1, ~67 kDa; EAAT2, ~70 kDa; EAAT3, ~65 kDa; EAAT4, ~69 kDa; EAAT5, ~68 kDa. Cyclophilin B was used as a protein loading control. Blots are shown as a representative of two replicate experiments.
